# Small-molecule agonist AdipoRon alleviates diabetic retinopathy through the AdipoR1/AMPK/EGR4 pathway

**DOI:** 10.1186/s12967-023-04783-3

**Published:** 2024-01-02

**Authors:** Yihan Wang, Yujuan Liu, Junwei Fang, Xindan Xing, Hanying Wang, Xin Shi, Xinyi Liu, Tian Niu, Kun Liu

**Affiliations:** 1 Department of Ophthalmology, Shanghai General Hospital, Shanghai Jiao Tong University School of Medicine, Shanghai, 200080 China; 2 National Clinical Research Center for Eye Diseases, Shanghai, 200080 China; 3 Shanghai Key Laboratory of Ocular Fundus Diseases, Shanghai, 200127 China; 4 Shanghai Engineering Center for Visual Science and Photomedicine, Shanghai, 200080 China; 5 Shanghai Engineering Center for Precise Diagnosis and Treatment of Eye Disease, Shanghai, 200080 China; 6 Department of Ophthalmology, Shanghai Renji Hospital, School of Medicine, Shanghai, 200127 China

**Keywords:** Diabetic retinopathy, AdipoRon, Oxidative stress, Early growth response factor 4

## Abstract

**Background:**

Diabetes mellitus (DM) is a progressive disease that involves multiple organs due to increased blood glucose, and diabetic retinopathy (DR) is the main complication of DM in the eyes and causes irreversible vision loss. In the pathogenesis of diabetic vascular disease, oxidative stress caused by hyperglycemia plays an important role in Müller cell impairment. In recent years, AdipoRon, an adiponectin analog that demonstrated important physiological functions in obesity, diabetes, inflammation, and cardiovascular diseases, demonstrated cellular protection from apoptosis and reduced inflammatory damage through a receptor-dependent mechanism. Here, we investigated how AdipoRon reduced oxidative stress and apoptosis in Müller glia in a high glucose environment.

**Results:**

By binding to adiponectin receptor 1 on Müller glia, AdipoRon activated 5ʹ adenosine monophosphate-activated protein kinase (AMPK)/acetyl-CoA carboxylase phosphorylation downstream, thereby alleviating oxidative stress and eventual apoptosis of cells and tissues. Transcriptome sequencing revealed that AdipoRon promoted the synthesis and expression of early growth response factor 4 (EGR4) and inhibited the cellular protective effects of AdipoRon in a high-glucose environment by reducing the expression of EGR4. This indicated that AdipoRon played a protective role through the EGR4 and classical AMPK pathways.

**Conclusions:**

This provides a new target for the early treatment of DR.

**Graphical Abstract:**

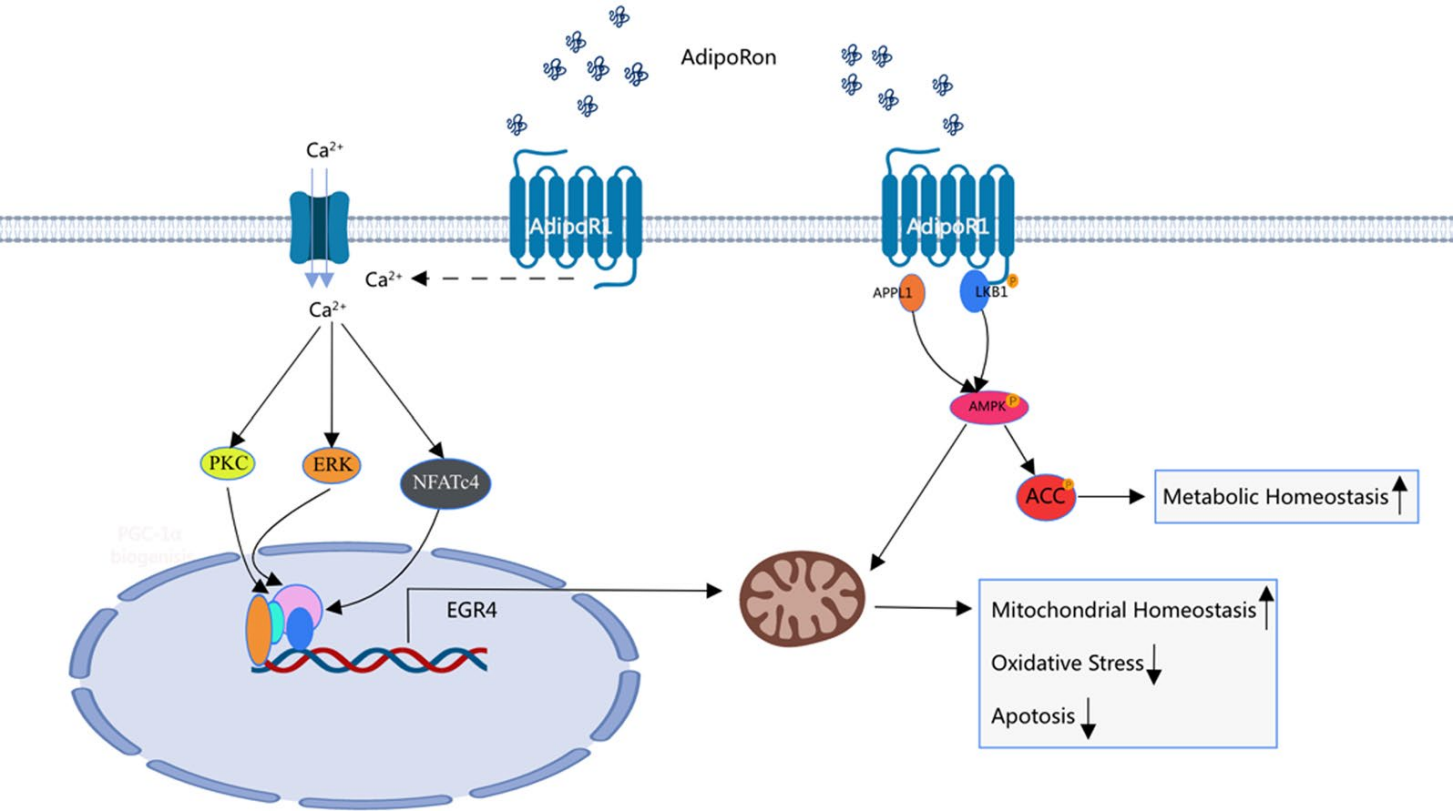

**Supplementary Information:**

The online version contains supplementary material available at 10.1186/s12967-023-04783-3.

## Background

The release of the 10th edition of the Global Diabetes Map by the International Diabetes Federation revealed a critical global trend toward a diabetes epidemic. Approximately 537 million adults worldwide have diabetes (1 in 10 have diabetes), which is expected to rise to 783 million in the next 15 years. Among these, those affected by diabetic retinopathy (DR) are expected to rise to 191 million by 2030, and DR is emerging as one of the leading causes of blindness globally. The overall prevalence of any form of DR is estimated to be approximately 27%, consisting of 4.6% diabetic macular edema, 25.2% non-proliferative DR, and 1.4% proliferative DR (PDR) [1–4]. Although the control of glucose is an important method to avoid complications, continuous hyperglycemia will trigger the legacy effect in the body, and complications will continue to develop even after the stable control of glucose [5, 6].

The lack of effective treatments necessitates an in-depth study of the underlying mechanisms. Oxidative stress is one of the factors involved in DR [7, 8]. Long-term hyperglycemia leads to an imbalance between reactive oxygen species (ROS) production and consumption in cells. In turn, the excessive accumulation of ROS aggravates the disorder of glucose metabolism [9]. Finally, it causes mitochondrial damage, inflammation, apoptosis, and structural and functional abnormalities in the retina [10–12]. Therefore, it is necessary to elucidate the molecular mechanisms underlying the progression of DR.

In the 1990s, researchers discovered that leptin and adiponectin could reactivate and help reshape adipose tissue, transforming it from a simple energy reservoir into a complex and highly active endocrine organ [13, 14]. Hyperlipidemia undoubtedly aggravates microtubule complications of diabetes. Attention is being paid to these adipokines that were overlooked in previous studies. Adiponectin, an adipocyte-specific factor, was first identified in 1995. Over the past two decades, numerous studies have elucidated the physiological functions of adiponectin in obesity, diabetes, inflammation, atherosclerosis, and cardiovascular disease [15, 16]. In 2013, Professor Okada-Iwabu [17] identified a small-molecule agonist adiponectin analog that activated adiponectin receptor (AdipoR)1/2.AdipoRon,like adiponectin,works by binding to specific receptor AdipoR1(primarily expressed in muscle and fat cells)and AdipoR2(expressed in hepatocytes), playing an important role in regulating glucose and lipid metabolism, inflammation and oxidative stress in the body [18].It stimulates mitochondrial biosynthesis and inhibits inflammation by increasing glucose and Non esterified fatty acids (NEFA) utilization [17]. Zhang [19] found that,both in vivo and vitro, AdipoR1 signaling restored SIRT3-mediated mitochondrial homeostasis, played a protective role against oxidative injury in traumatic brain damage.As an important regulator of lipid and glucose metabolism, AdipoR1 had intrinsic ceramidase activity, deficiency in which was found ceramide accumulation in the retina in AdipoR1^−/−^ mice leading to the death of photoreceptors [20]. During the retinal vascularization of infants,AdipoRon, possibly through targeting different retinal neurons, controlled retinal vascular formation [21],demonstrating intricate functional coupling of AdipoR1 between neurons, glial cells, and blood vessels. In addition to alleviating oxidative stress, AdipoRon also significantly increased autophagosome clearance, reduced infarct size and improved cardiac function in MI mice by stimulating autophagosome formation [22]. These all indicated that AdipoRon,as an excellent potential drug,involved different mechanisms in different tissue cells [23, 24]. AdipoRon showed a protective effect against hyperglycemia-induced renal tissue damage in DB/DB mice [25]. Moreover, it reduces neuronal mortality, increases adenosine triphosphate production, inhibits ROS production, and activates pathways downstream of AdipoR1, such as AMPK, PPARα, peroxisome proliferator-activated receptor-gamma coactivator (PGC) 1, nuclear respiratory factor, ultimately improving mitochondrial function and other related pathological processes [26]. Previous studies have shown that the expression of both adiponectin and AdipoR1 is increased in the retinas of patients with diabetes [27]. Moreover, the concentration of aqueous adiponectin in patients with PDR is significantly higher than that in patients without diabetes [28]. These studies suggest that AdipoR1 activation may represent a protective compensatory mechanism to alleviate DR [29, 30].

In our study, we confirmed the protective effect of AdipoRon against DR after regular administration to streptozotocin (STZ)-induced diabetic mice. These effects included reduced apoptosis of retinal ganglion cells (RGC), recovery of electroretinographic recordings, and reduction of oxidative stress and apoptosis of the retina. The protective effect was verified in Müller cells cultured with high glucose in vitro. When further exploring the mechanism of protection, we observed that early growth response factor 4 (EGR4) may reduce cell death by protecting cells from oxidative stress.

## Methods

### Animals

Male C57BL/6j mice (*n* = 60) weighing 20–22 g were purchased from Cavens Biogle (Suzhou, China) Model Animal Research Co. Ltd. (Jiangsu, China). All the animals were fed in a pathogen-free environment, kept at a constant temperature of 22 ± 1 °C for a 12 h day-night cycle. The mice had free access to water and food. All animal experiments were approved by the Ethics Committee of Shanghai General Hospital and conducted following the animal care standards set forth by the National Institutes of Health.

### Animal feeding and modeling

All 4 week mice (*n* = 60) were randomly divided into four groups of 15 mice each, and a 45% high-fat diet was given to induce insulin resistance. After 1 month, three groups of four mice were randomly selected to receive an intraperitoneal injection of STZ (40 mg/kg) for five consecutive days. All mice were fed under equal conditions for the following 3 months.


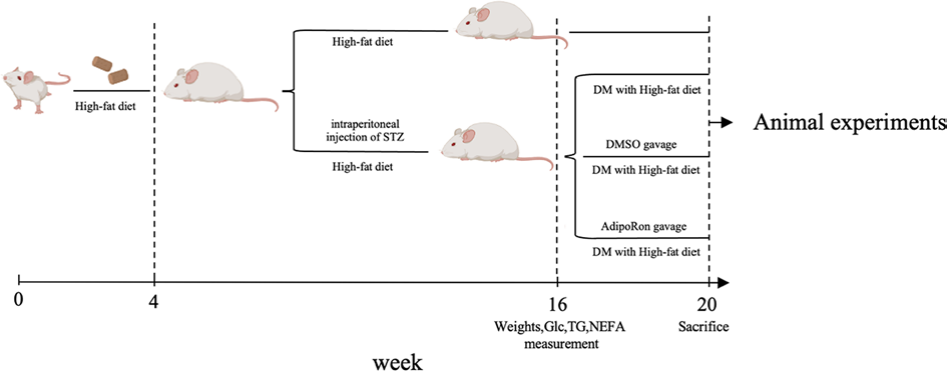


### Reagents and antibodies

The following reagents were used: Streptozotocin (Merck, Darmstadt, Germany); AdipoRon (Selleckchem, Houston, TX, USA); a triglyceride assay kit and a non-esterified free fatty acid assay kit (Jiancheng Bioengineering Institute, Nanjing, China); and dihydroethidium (Thermofisher, MA, USA). The following antibodies were used: Rabbit monoclonal to AdipoR1 (EPR6626,Abcam, Cambridge, UK); rabbit polyclonal to RNA-binding protein with multiple splicing (RBPMS; Abcam); rabbit monoclonal to glutamine synthetase (EPR13022(B),Abcam); chicken polyclonal to glial fibrillary acidic protein (GFAP; Abcam); rabbit polyclonal to EGR4 (Abcam); phospho-AMPKα rabbit monoclonal antibody (mAb) and AMPKα rabbit mAb (40H9/D5A2,Cell signaling, Boston, USA); phospho-acetyl CoA carboxylase (ACC) rabbit mAb and ACC rabbit mAb (D7D11/C83B10,Cell signaling); cleaved caspase-3 rabbit mAb (Cell signaling), β-actin (8H10D10,Cell signaling); donkey anti-rabbit AlexaFluor-594 and donkey anti-chicken Alexa 488 (Jackson ImmunoResearch, Philadelphia, USA). The following reagents were used: 5,6-chloromethyl-2ʹ,7ʹ-dichlorodihydrofluorescein diacetate (CM-H_2_DCFDA; Thermofisher); RNAimax (Thermofisher); Opti-MEM I (Thermofisher); small interfering RNA (siRNA; GenePharma, Shanghai, China); Cell Counting Kit-8 (CCK-8; Dojindo, Tokyo, Japan); and 4ʹ,6-diamidino-2-phenylindole (DAPI; Absin, Shanghai, China).

### Basic information and measurement of plasma glucose and lipids

Fasting body weight was measured in four groups of mice three months after the successful modeling of diabetes. Blood samples were left standing for 60 min before centrifugation at room temperature for 10 min at 635 g which were collected via submandibular vein puncture.Then,the supernatant was removed by aspiration for plasma glucose(Glc), triglyceride(TG), nonestesterified fatty acid (NEFA) meaturements.After that, a group of DM mice were conducted AdipoRon gavage, and DMSO gavage was set up as the control group at the same time. Fasting weight of all mice was repeated one month after oral administration. Blood samples were drawn by methods mentioned before for Glc, TG, NEFA measurements. All operations were completed in accordance with the manufacturer’s instructions.

### Oral administration

As all mice were continuously fed a high-fat diet, the four groups were named high-fat diet (HFD), HFD + diabetes mellitus (DM) control (DM), HFD + DM + dimethyl sulfoxide (DMSO) negative (DMSO), and HFD + DM + AdipoRon (AdipoRon). AdipoRon dissolved in 5% DMSO was suspended in 0.5% carboxymethylcellulose and administered orally to the AdipoRon group (*n* = 15) at a 30 mg/kg/day dose for 28 days [17, 25].

The DMSO group was administered an equal volume of DMSO (negative control) via oral gavage. The remaining mice were simultaneously fed.

### Electroretinogram (ERG)

After a minimum of 12 h of complete darkness, the mice were anesthetized with sodium pentobarbital. Following corneal anesthesia and mydriasis, reference and ground electrodes were inserted into the skin of the forehead and tail, respectively. Corneal electrodes were placed on the surface of the cornea using a lubricant eye gel. The Phoenix Ganzfeld System (Phoenix Research Labs, Pleasanton, CA) was applied to record the scotopic a/b-wave at a stimulus intensity of 1.0–4.0 log^10^ (cd·sec/m^2^).

### Retina ganglion cell (RGC) counting

A method for assessing the number of retinal ganglion cells.The mice were euthanized by cervical dislocation, and their retinas were completely removed and fixed in 4% paraformaldehyde at room temperature for 1 h. After sufficient washes in phosphate-buffered saline (PBS), blocking in PBS containing 5% bovine serum albumin (BSA) for 30 min followed. Subsequently, the samples were incubated in a 4 ℃ shaker overnight after diluting with RBPMS at 1:200. The following day, the retina was cleaned three times in PBS and incubated at room temperature with a diluted secondary antibody (1:500) for 1 h. Using the optic nerve as the center, the retina was cropped radially into four-leaf clover shapes, laid flat on a slide, sealed, and fixed using an anti-fluorescence quencher. A confocal microscope was used for observation.

### Retina ROS content detection

Dihydroethidium (DHE) is a common fluorescent probe used for the detecting superoxide anion levels [25]. According to the manufacturer's instructions, superoxide production in the retina was expressed by relative fluorescence intensity.The probe was added to the section immediately and incubated with 0.1 μmol/L PBS. After incubation at 37 °C for half an hour, sections were sealed with the anti-fluorescence quencher. Fluorescent images were obtained using a confocal microscope (594 nm).

### The reactive gliosis of Müller glia

After thawing to room temperature, the sections were washed three times with PBS every 5 min. Blocking solution (1% triton and 5% BSA in PBS) was added for 30 min at 37 °C, and the sections were incubated with anti-GFAP (1:200 dilution) overnight at 4 °C. The sections were washed and incubated with a secondary antibody (1:500) for 60 min, protected from light at room temperature. 5 min after DAPI staining, the sections were sealed with an anti-fluorescence quencher. Fluorescence images were obtained using a confocal microscope.

### Western blot assay for protein expression

Proteins extracted from the retina were subjected to sodium dodecyl-sulfate polyacrylamide gel electrophoresis and transferred to nitrocellulose membranes. Targeted membranes were blocked in 5% BSA for 60 min at 37 °C followed by incubation with the corresponding primary antibody (1:1000) diluted in Tris-buffered saline with Tween 20 (TBST) overnight at 4 °C. On the second day, the membranes were sufficiently cleaned and incubated with a secondary antibody (1:2000) diluted in TBST for 1 h at room temperature. The immunoreactive bands were detected using a chemiluminescent substrate.

### Primary Müller glia extraction

The following materials were used: PBS, penicillin–streptomycin (P/S), Dulbecco’s Modified Eagle Medium (DMEM; full and FBS-free), trypsin, dishes, sterilized surgical instruments, pipettes, 75% alcohol, centrifuge tubes, Eppendorf tubes, and waste liquid boxes. The cells were obtained from 5 to 7 day-old C57 mice pups. Before removal of the eyeballs, pups were soaked in 75% alcohol for complete disinfection. The removed eyeballs were placed in a 6 cm dish, washed at least three times with PBS + P/S to remove excess tissue, and immersed and incubated in DMEM for 1 days at 37 °C. After rinsing with PBS + P/S, the eyeballs were placed in a round-bottom Eppendorf tube containing 0.25% trypsin and digested in a 37 °C incubator for half an hour. The digested eyeball was then transferred to a 6 cm dish, and a full medium was added to terminate digestion. The retina was carefully extracted from the dish, collected in a 15 mL centrifuge tube, and centrifuged at 1500 rpm for 5 min. Subsequently, the supernatant was discarded, the full medium was added, and the suspension was gently blown. The suspension was then uniformly seeded in a dish and grown in a regular incubator.

### CCK-8

AdipoRon was dissolved in DMSO according to the manufacturer’s instructions, and a 1-mM stock concentration was prepared. The agonist was diluted from the maximum concentration of 100 mM with a 1/twofold gradient dilution to a total of six concentrations. The cells were cultured with 100 μM, 50 μM, 25 μM, 12.5 μM, 6.25 μM, and 3.125 μM in 96-well plates. Six parallel holes were set up for each concentration, and CCK-8 solution (10 μL) was added to each well when the cells were fused to 70–80%. The entire process was conducted in the dark. After 1–4 h of incubation, absorbance was measured using a microplate reader at 450 nm.

### Transfection of siRNA

The siRNAs, blank siRNA (negative control), and AdipoR1-specific siRNA (Gene ID: 72,674) were complexed with the transfection reagent according to the manufacturer’s instructions. The sequences of the siRNAs were as follows: AdipoR1; sense 5ʹ—TCTTCGGGATGTTCTTCCTGG—3ʹ and antisense 5ʹ—TTGGAAAAAGTCCGAGAGACC—3ʹ. Müller glia was treated with 12.5 μM AdipoRon in high-glucose, cultured in six-well plates, and transfected with AdipoR1 (25 pmol) and blank siRNA using RNAimax in Opti-MEM (R) I reduced serum medium for 48 h. The medium was replaced, and then an AdipoRon concentration of 12.5 μM was maintained. Cells were prepared for the following tests:

### Quantification of total cellular ROS levels

Method 1: The intracellular ROS levels were measured using CM-H_2_DCFDA(Invitrogen). The cells (1 × 10^4^) were inoculated in 96-well plates and cultured in low glucose and high glucose and were treated with DMSO,AdipoRon,NC-siRNA and AdipoR1-specific siRNA. After incubation for 48 h, cells were washed with PBS, followed by adding 10 μM CM-H_2_DCFDA to each well. The cells were then incubated at 37 °C for 30 min, and the plates were observed under a fluorescence intensity reader.

Method 2: The cells (5 × 10^4^) were inoculated in 6-well plates and cultured in high glucose + AdipoRon environment with short hairpin RNA (EGR4-specific shRNA). After infection for 72 h, cells were washed with normal saline (0.9%), followed by adding 10 μM of 2ʹ—7ʹdichlorofluorescin diacetate (DCFH-DA; Beyotime) to each well. The cells were then incubated at 37 °C for 30 min, carefully digested, washed, and resuspended in normal saline (0.9%) for flow cytometry (Beckman).

### Transcriptome resequencing

The cells were inoculated in 3.5 cm dishes. After corresponding treatments, cellular RNA was extracted using an RNA extraction kit according to the manufacturer’s instructions. RNA purity and quantification were evaluated using a NanoDrop 2000 spectrophotometer, and RNA integrity was assessed using the Agilent 2100 Bioanalyzer. Subsequently, libraries were constructed using the TruSeq Stranded mRNA LT Sample Prep Kit (Illumina, San Diego, CA, USA) according to the manufacturer’s instructions.

The libraries were sequenced on an Illumina HiSeq X Ten platform, generating 150-bp paired-end reads. An average of 14,000 raw reads from each sample were examined. The FASTQ format was first processed using Trimmomatic to acquire raw data (raw reads) [31] and clean reads were obtained, after which approximately 130 clean reads for each sample were retained and mapped to the human genome (GRCh38) using HISAT2 [32]. The fragments per kilobase per million mapped fragments [33] of each gene were calculated using Cufflinks [34], and the read counts of each gene were obtained using HTSeq- count [35]. Differential expression analysis was performed using the DESeq R package (2012). The threshold for significantly differential expression was set at *p* < 0.05 and foldchange > 2 or < 0.5.

### Lentivirus-shRNA transfection

The lentivirus ad-shEGR4, expressing a shRNA targeting mouse EGR4 (GenBank NM_020596.3), and vehicle control shRNA were purchased from OBiO Technology (Shanghai, China). The shRNAs were mixed before transfection according to the manufacturer’s instructions. The knockdown target sequences are shown below 5ʹ—GCACAGCAAGGTGCACCTAAATTTAGGTGCACCTTGCTGTGC—3ʹ.

### Measurement of mitochondrial ROS (mtROS)

The mtROS were detected using the MitoSOX red mitochondrial superoxide indicator (Invitrogen). Cells (5 × 10^4^) were inoculated in 6-well plates and cultured in high glucose + AdipoRon environment with short hairpin RNA (EGR4-specific shRNA and NC). Harvested cells were washed three times and then resuspended in Hanks’ balanced salt solution (HBSS) buffer containing 5 μM MitoSOX at 37 °C for 20 min. After washing with HBSS buffer, the mtROS levels were quantified using flow cytometry (Beckman).

### Statistical analysis

The normality and variance homogeneity assumptions of the data were assessed before analysis. Shapiro–Wilk and Levene’s tests were conducted for normal distribution and homogeneity of variance. All the data were random, independent, and normally distributed. Experimental results were represented as mean ± standard deviation (SD), and a one-way ANOVA with post-hoc comparisons using Dunnett's test was used to compare multiple groups. Differences were considered statistically significant at *p* < 0.05 (*p < 0.05, **p < 0.01, and ***p < 0.001).

## Results

### Fasting body weight, plasma glucose, triglycerides (TG), and non-esterified free fatty acids (NEFA) of mice

**Fig. 1:**
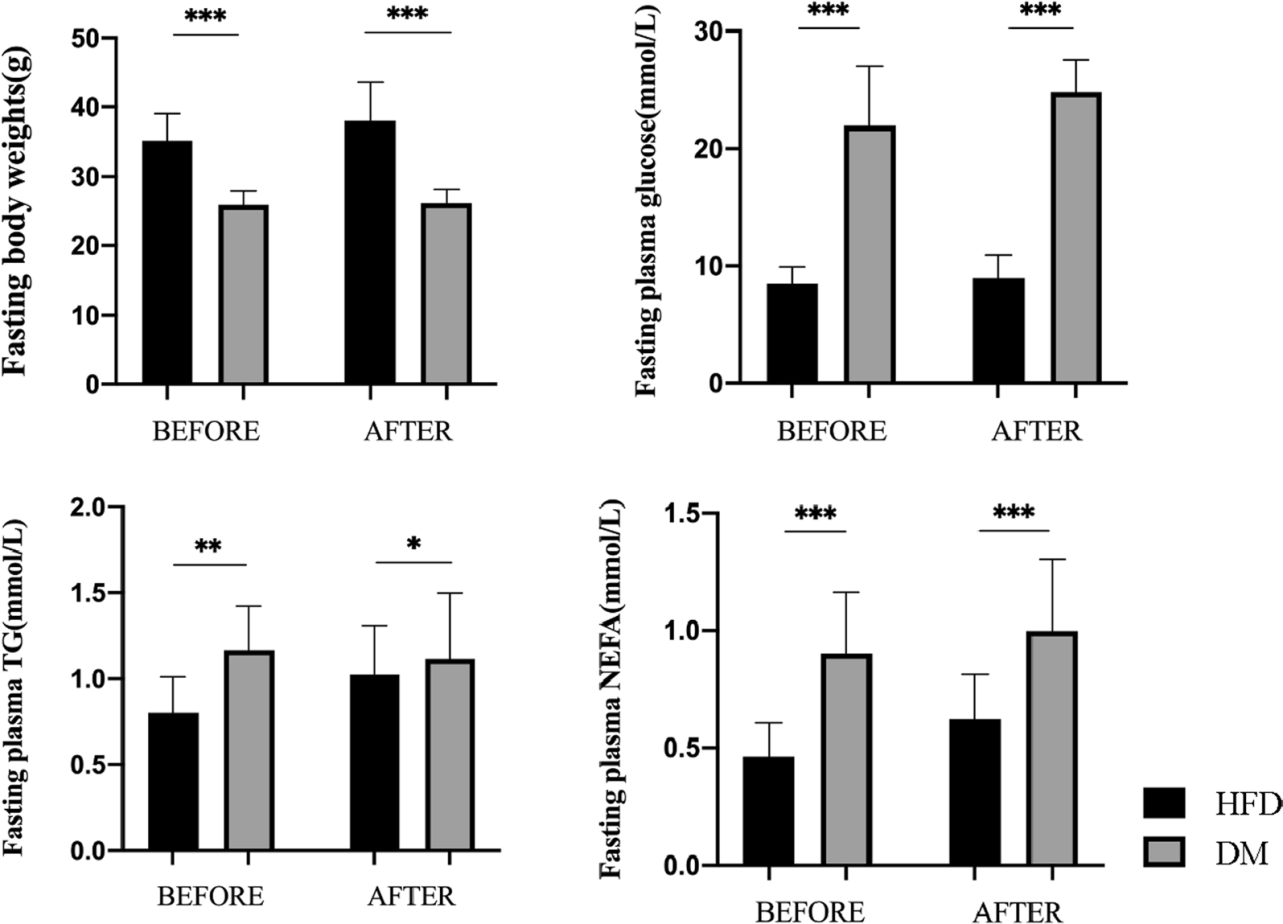
Comparison of basic information between HFD, DM before and after gavage period (These two groups of mice were not given intragastric treatment and were only fed as controls). The results are shown as mean ± standard deviation, *p < 0.05, **p < 0.01, ***p < 0.001. HFD, high-fat diet; DM, high-fat diet + diabetes mellitus control

After the modeling of diabetes, fasting plasma glucose, TG, and NEFA levels in the DM group were significantly higher than those in the HFD group (Fig. 1) (Table 1), indicating that diabetes causes elevated plasma glucose and abnormal lipid metabolism. Compared with that in the HFD group, the weight of the other three groups decreased significantly, which was consistent with the symptoms of diabetes.

**Table 1:** Body weights and plasma glucose, triglyceride (TG), and non-esterified free fatty acid (NEFA) levels of four groups of mice before and after gavage

Characteristic	HFD	DM	DMSO	AdipoRon
Fasting body weights				
Before	35.07 ± 3.95	25.94 ± 2.02	24.75 ± 1.91	25.48 ± 2.19
After	38.15 ± 6.04	26.19 ± 1.97	25.31 ± 1.03	24.41 ± 1.89
*P*-value	< 0.05	/	/	/
Fasting plasma glucose				
Before	8.37 ± 1.45	24.85 ± 2.62	22.38 ± 4.63	23.69 ± 3.31
After	8.81 ± 1.86	24.78 ± 2.78	23.43 ± 3.24	24.27 ± 2.33
*P*-value	/	/	/	/
Fasting plasma TG				
Before	0.80 ± 0.23	1.16 ± 0.26	1.03 ± 0.38	1.07 ± 0.34
After	1.03 ± 0.30	1.12 ± 0.38	1.08 ± 0.28	0.95 ± 0.28
*P*-value	< 0.05	/	/	/
Fasting plasma NEFA				
Before	0.46 ± 0.14	0.90 ± 0.26	0.76 ± 0.23	0.88 ± 0.24
After	0.62 ± 0.19	1.00 ± 0.30	0.90 ± 0.43	0.92 ± 0.27
*P*-value	< 0.05	/	/	/

*HFD* high-fat diet, *DM* high-fat diet + diabetes mellitus control, *DMSO* high-fat diet + diabetes mellitus + dimethyl sulfoxide negative control, AdipoRon, high-fat diet + diabetes mellitus + AdipoRon. The results are represented as mean ± standard deviation

Without any intervention, the weight was significantly greater in the HFD mice than that of the diabetic mice for the same period after 4 months. At the same time, the levels of glucose, TG and NEFA were generally lower than that of diabetic mice (Fig. 1).

**Fig. 2:**
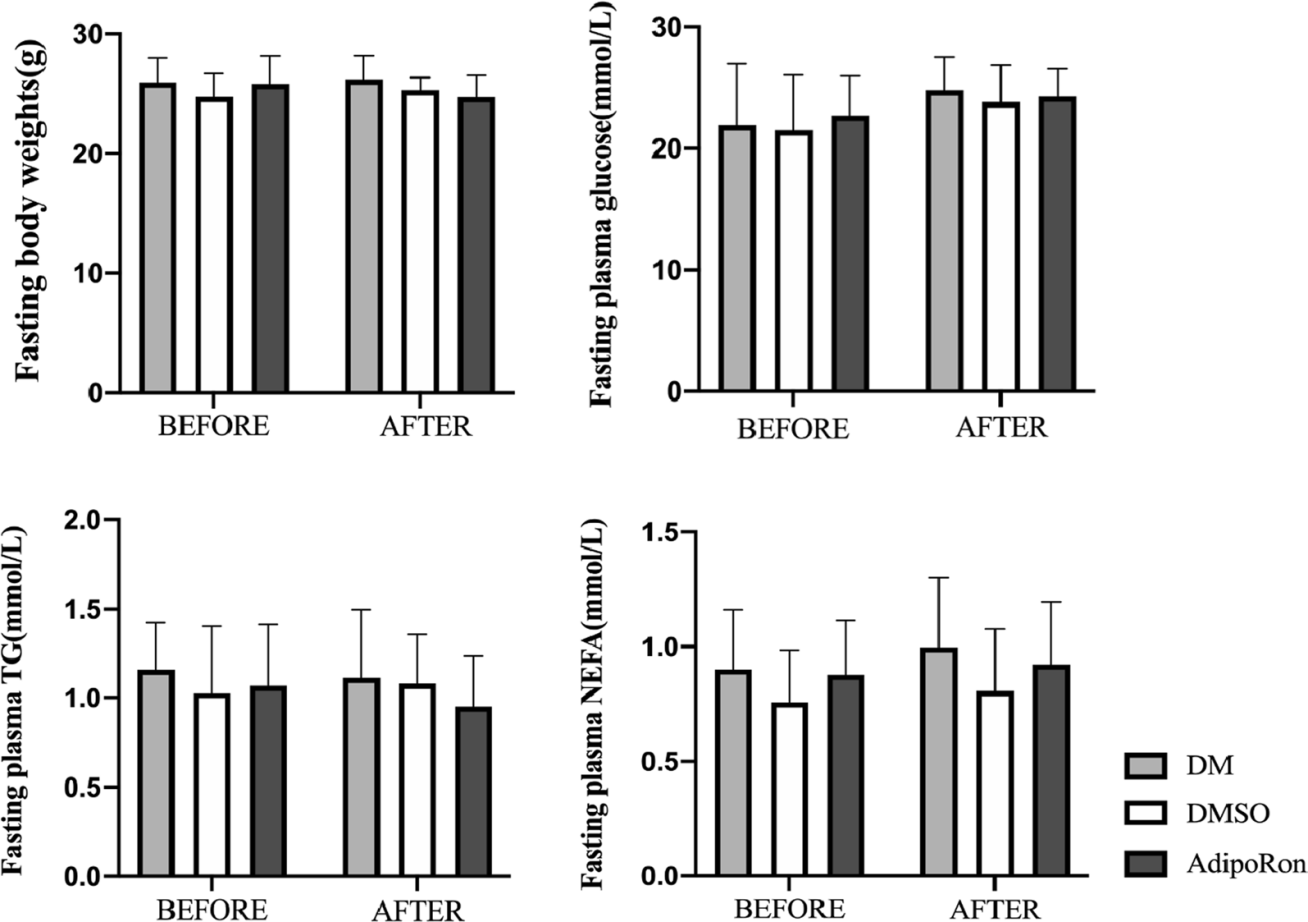
Comparison of the basic information of the DM, DMSO, and AdipoRon groups before and after intragastric administration.After a group of diabetic mice received AdipoRon for 4 weeks, there were no changes in fasting body weight, glucose, TG and NEFA before and after administration in the three diabetic mice groups.The results are shown as mean ± standard deviation, * p < 0.05. DM, high-fat diet + diabetes mellitus control; DMSO, high-fat diet + diabetes mellitus + dimethyl sulfoxide negative control; AdipoRon, high-fat diet + diabetes mellitus + AdipoRon

To observe whether AdipoRon had an effect on fasting plasma glucose,lipids,or weights of diabetic mice.We compared the related indicators among three groups before and after intragastric administration.However, a pairwise comparison of the DM, DMSO, and AdipoRon-treated groups showed no significant changes in neither body weight nor plasma glucose, TG, and NEFA levels after gavage, indicating that AdipoRon had no significant influence on long-term glucose and lipid homeostasis (Fig. 2).

### The examinations of electroretinogram (ERG)

**Fig. 3:**
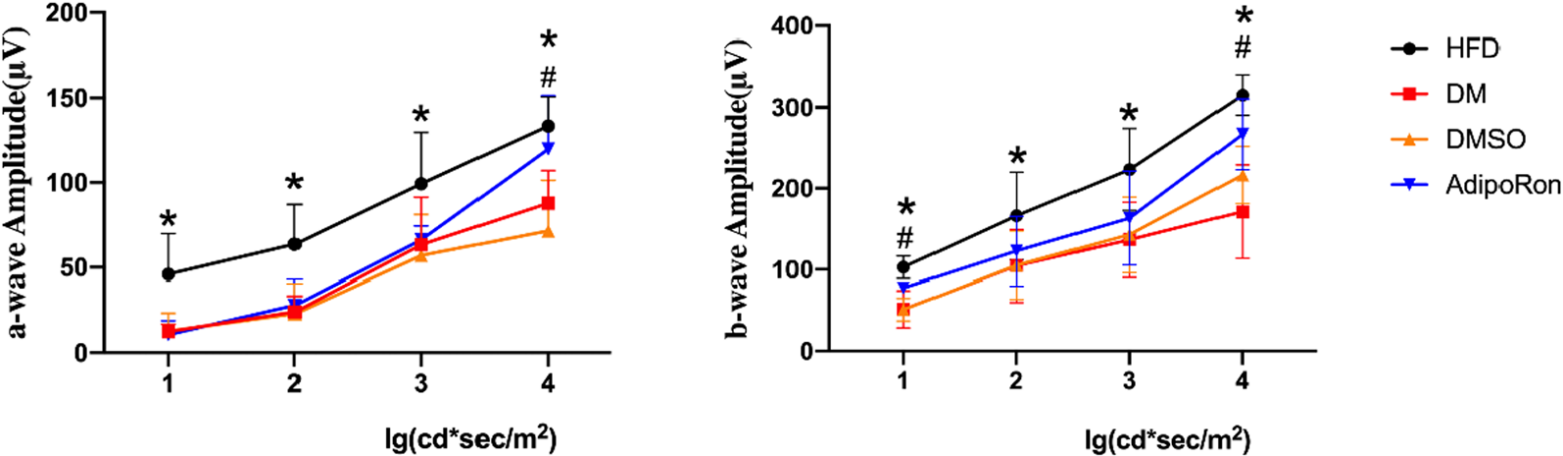
Electroretinogram (ERG) change of four groups. ERG a-wave is shown on the left, and ERG b-wave is on the right.The stimulation intensity increased along the right gradient of the x-axis. The amplitude of the four intensity cut-off points is shown. The amplitudes of a-wave and b-wave of diabetic mice were decreased compared with HFD mice and AdipRon reversed the change under high stimulation (intensity 4).Data are represented as the mean ± standard deviation. *means of HFD vs. DM, *p* < 0.05; ^#^means of AdipoRon vs. DM, *p* < 0.05. HFD, high-fat diet; DM, high-fat diet + diabetes mellitus control; DMSO, high-fat diet + diabetes mellitus + dimethyl sulfoxide negative control; AdipoRon, high-fat diet + diabetes mellitus + AdipoRon

The b-wave represented the physiological electrical activity of Müller cells.Upon gradient stimulation, the a-wave amplitude in the HFD group was high, whereas that in the DM and DMSO groups remained low. After AdipoRon treatment, the a-wave recovered significantly under the increased stimulation (intensity 4), and the difference was statistically significant compared with that of the DM and DMSO groups. and it showed the same tendency upon the same gradient stimulation. The amplitude in the HFD group was higher, whereas that in the DM and DMSO groups remained low. After AdipoRon treatment, the b-wave recovered to a certain extent under low stimulation (intensity 1) and high stimulation (intensity 4), which was statistically significant compared with that of the DM and DMSO groups (Fig. 3). This suggests that AdipoRon exerts a protective effect on the retina.

### The density of RGCs

**Fig. 4:**
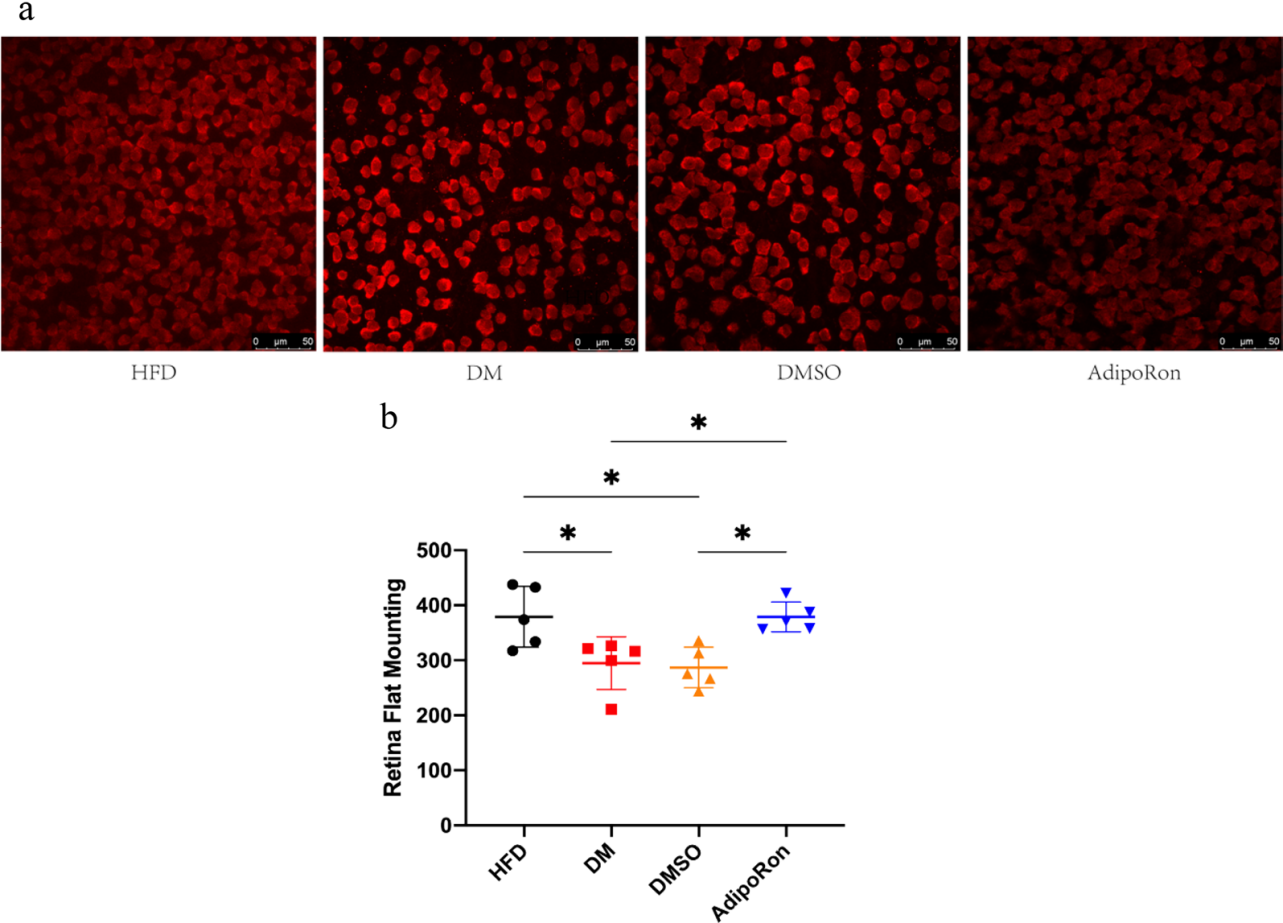
**a**. RNA-binding protein with multiple splicing (RBPMS) staining (red) of the retinal flat mount in each group.After treated with AdipoRon, the number of RGCs recovered significantly, and the density was similar to that of the HFD group. **b**. Retinal ganglion cell (RGC) quantity in retinal tissues of mice in four groups. Data are represented as the mean ± standard deviation * *p* < 0.05. HFD, high-fat diet; DM, high-fat diet + diabetes mellitus control; DMSO, high-fat diet + diabetes mellitus + dimethyl sulfoxide negative control; AdipoRon, high-fat diet + diabetes mellitus + AdipoRon

RBPMS can specifically label ganglion cells [36, 37]. RGCs in healthy people are dense and evenly distributed. In contrast, in people with diabetes, high glucose levels accelerate the apoptosis of RGC cells. Therefore, RGCs in the DM and DMSO groups were consistently lower than those in the HFD group, and this difference was statistically significant. After administration of AdipoRon, the number of RGCs recovered significantly, and the density was similar to that of the HFD group, with a statistically significant difference (Fig. 4). Our results showed that AdipoRon administration reduced the retinal RGCs loss in diabetic mice to a certain extent which was in agreement with the ERG findings outlined above.

### Retinal oxidative stress detection (DHE)

**Fig. 5:**
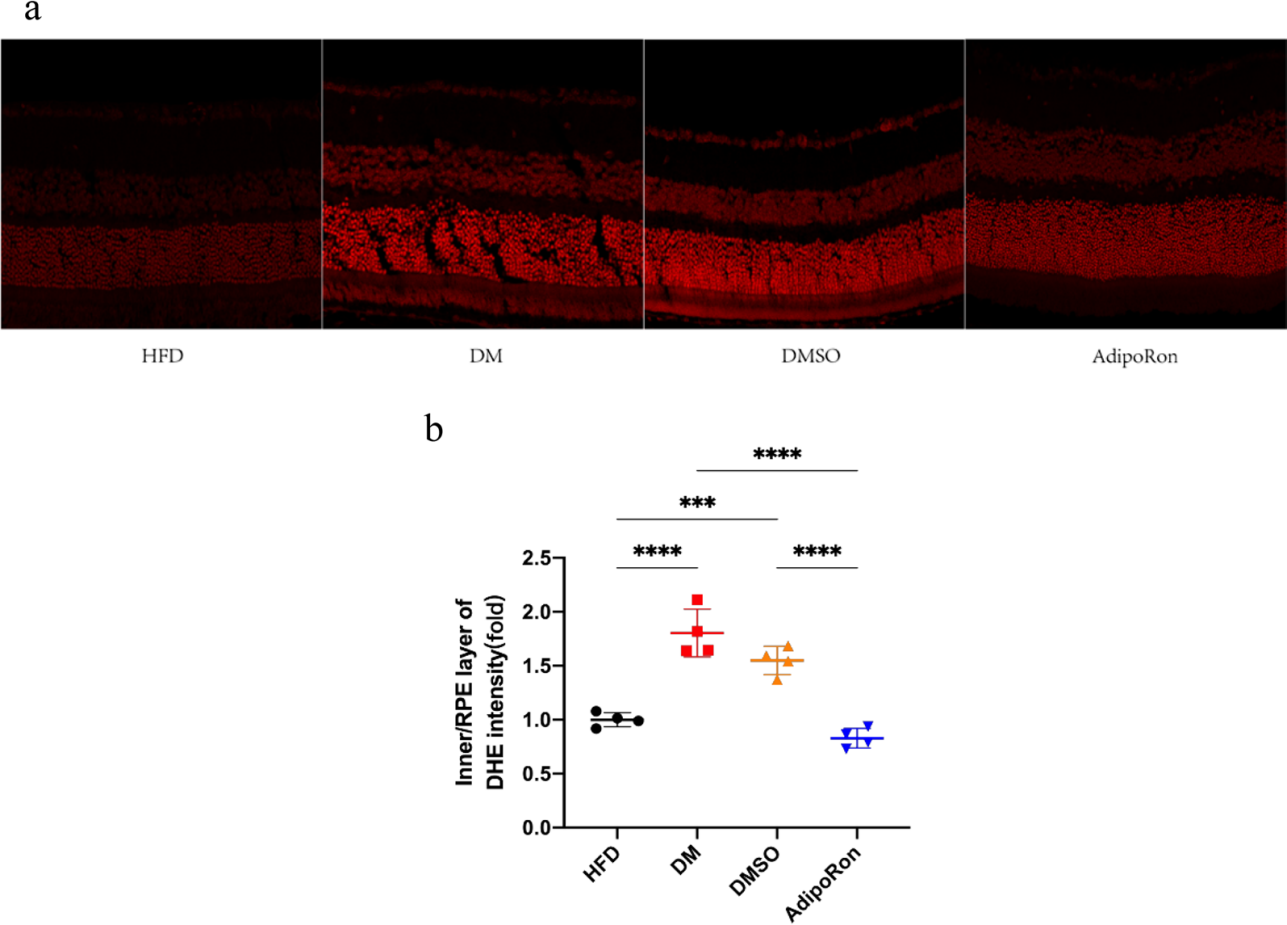
**a**. Measurement of oxidative stress in retinal tissue (red fluorescence). The ethidium bromide appears in higher level when superoxide anion produces more,especially in inner and RPE layers,leading to a stronger red fluorescence. **b**. Dihydroethidium (DHE) quantity in retinal tissues of mice in four groups. Data are represented as the mean ± standard deviation ***p < 0.001, ****p < 0.0001.HFD, high-fat diet; DM, high-fat diet + diabetes mellitus control; DMSO, high-fat diet + diabetes mellitus + dimethyl sulfoxide negative control; AdipoRon, high-fat diet + diabetes mellitus + AdipoRon

The superoxide indicator DHE is oxidized to form ethidium bromide which is then inserted into cell's DNA, causing the nucleus to stain bright fluorescent red [38–40]. Here we saw,the fluorescence intensity of the retina in the HFD group was weak, while that of the DM and DMSO groups was relatively enhanced indicating that the degree of retinal oxidative stress was increased.The attenuation of fluorescence intensity after AdipoRon gavage illustrated the oxidative stress level was significantly reduced (Fig. 5). These results suggested that AdipoRon may play a role in protection of the retinal physiological function by reducing oxidative stress,especially in inner and RPE layer.

### The reactive gliosis of Müller glia

**Fig. 6:**
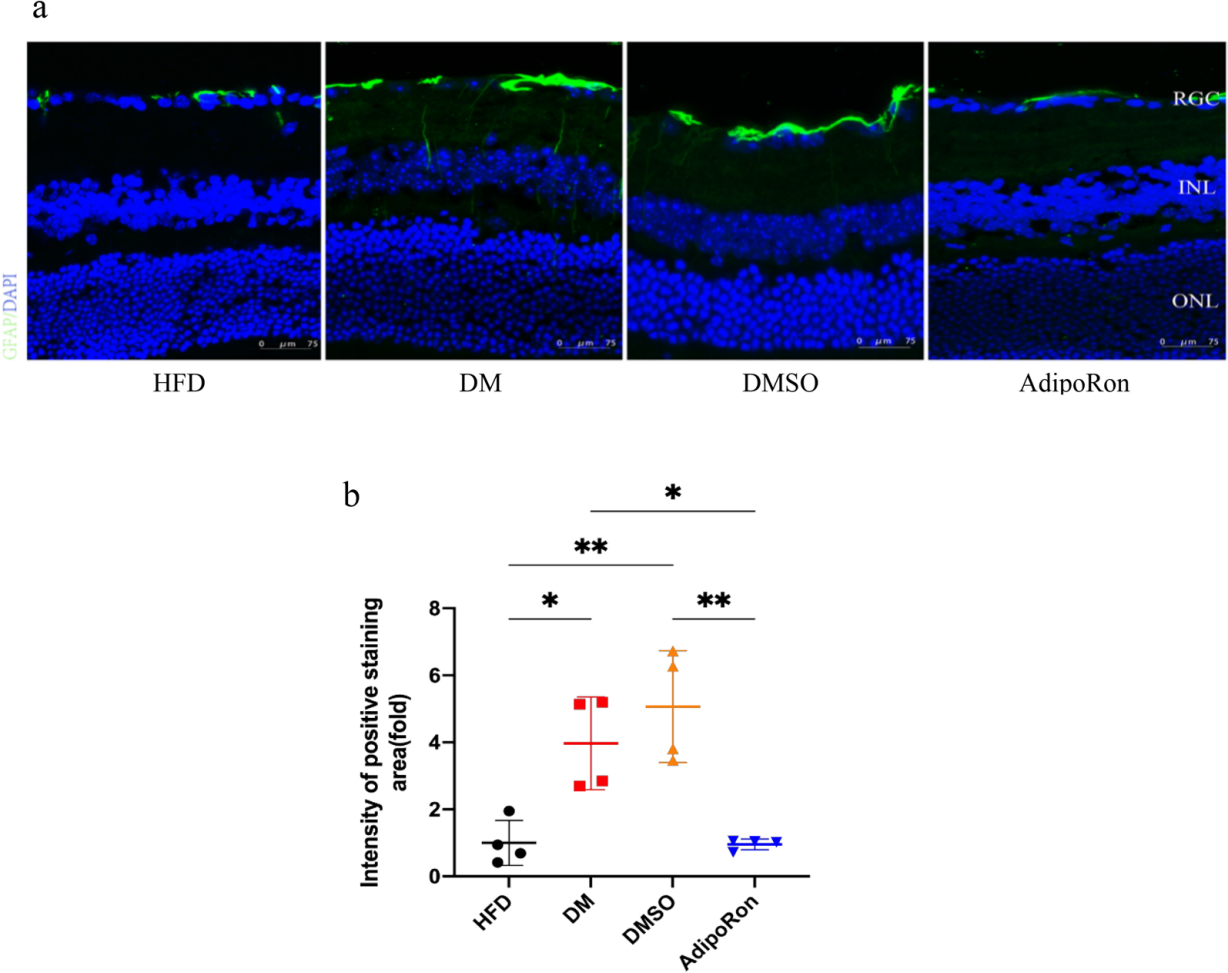
**a**. The reactive gliosis of Müller glia in each study group. GFAP staining of retinal cross sections (green) and nuclei counterstained with DAPI (blue) are shown. Here we can see vertically radial distribution after gliosis reactive,forming remarkable filamentous arrays in gliotic retina(DM,DMSO) **b**. Intensity of positive staining(green) quantity in retinal tissues of mice in four groups. Data are represented as the mean ± standard deviation *p < 0.05, **p < 0.01. RGC, retinal ganglion cell layer; *INL* inner nuclear layer, *ONL* outer nuclear layer, *GFAP* glial fibrillary acidic protein; DAPI, 4′,6-diamidino-2-phenylindole; *HFD* high-fat diet, *DM* high-fat diet + diabetes mellitus control; DMSO, high-fat diet + diabetes mellitus + dimethyl sulfoxide negative control; AdipoRon, high-fat diet + diabetes mellitus + AdipoRon

GFAP is mainly expressed in retinal astrocytes and Müller glia, and its activation reacts to the retinal pathological state. Early activation indicates that the retina starts actively repairing, and simultaneously, Müller glia secrete cytokines to accelerate the recovery from retinal injury. However, continuous activation damages the photoreceptors and ganglion cells. As shown in Fig. 6, GFAP protein (green signal) was mainly expressed on the vitreous side of the normal retina, and filamentous protein formation was rarely observed on the surface. The expression of GFAP increased in DM mice, and there was a whisker structure perpendicular to the vitreous surface of the retina that penetrated the inner plexus and core layer. The expression of GFAP increased in the DMSO group, and the same whisker structure was observed deep into the inner retina as that in the DM group. In contrast, the expression of GFAP in the AdipoRon group was significantly lower than that in DM and DMSO mice, and no whisker structure was observed. Therefore, the administration of AdipoRon can significantly reduce the reactive gliosis of Müller cells caused by diabetes, thus reducing retinal damage (Fig. 6).

### Altered protein expression of the retina

**Fig. 7:**
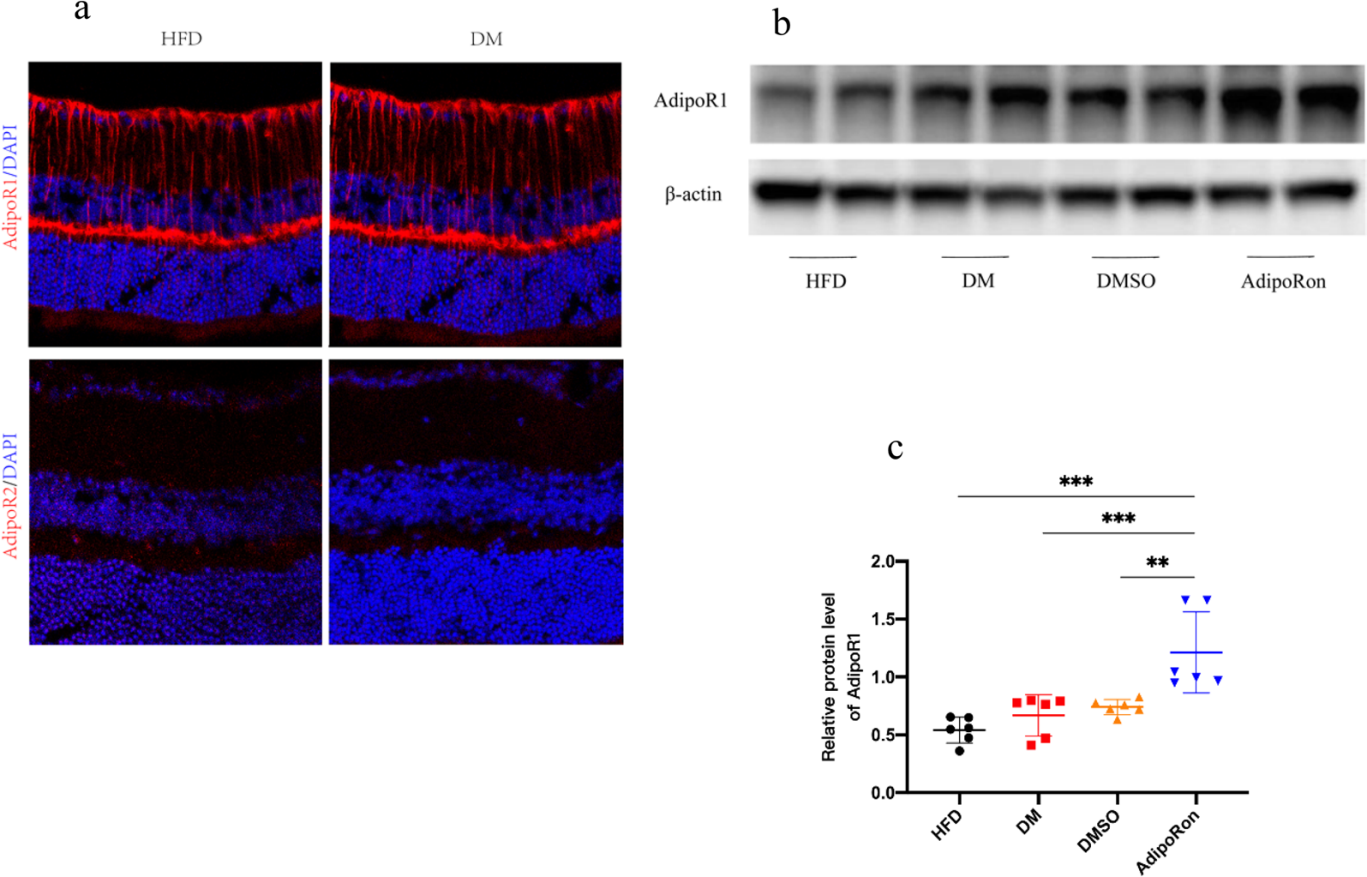
**a** Immunofluorescence verification of AdipoR1 in the retina on Müller glia and absence of AdipoR2 is shown. **b** Western blot verification of AdipoR1 slightly elevated in DM and significantly enhanced after AdipoRon administration. **c** Strips quantified using *Image* *J* software. The results are shown as mean ± standard deviation, ** p < 0.01, ***p < 0.001. AdipoR1, adioponectin receptor 1; DAPI, 4′,6-diamidino-2-phenylindole; HFD, high-fat diet; DM, high-fat diet + diabetes mellitus control; DMSO, high-fat diet + diabetes mellitus + dimethyl sulfoxide negative control; AdipoRon, high-fat diet + diabetes mellitus + AdipoRon

Adiponectin acts by binding to adiponectin receptors, namely, AdipoRs. We hypothesized that AdipoRon binds to AdipoRs in the retina to promote various physiological activities. We determined the distribution of receptors in the retina using immunofluorescence, thereby confirming the specific expression of AdipoR1 in Müller glia (Fig. 7a). Extracted proteins from the retina were then subjected to western blot analysis to observe and compare the differential expression of AdipoR1 and the changes in the downstream AMPK/ACC pathway proteins after activation of AdipoR1 among the four groups. As shown in Fig. 7b, the expression of AdipoR1 in the DM and DMSO groups was essentially the same or slightly elevated compared with that in the HFD group. Although previous studies have shown that plasma adiponectin levels are reduced in patients with diabetes, receptors may have compensatory increases to offset certain adverse effects of reduced adiponectin and maintain protective roles, which has been observed in a few studies on kidney injury after the administration of AdipoRon [41]. After the administration of AdipoRon, the expression of AdipoR1 was significantly increased, indicating that AdipoRon enhanced the expression of AdipoR1 in the retina. These differences were statistically significant (*p* < 0.05).

**Fig. 8:**
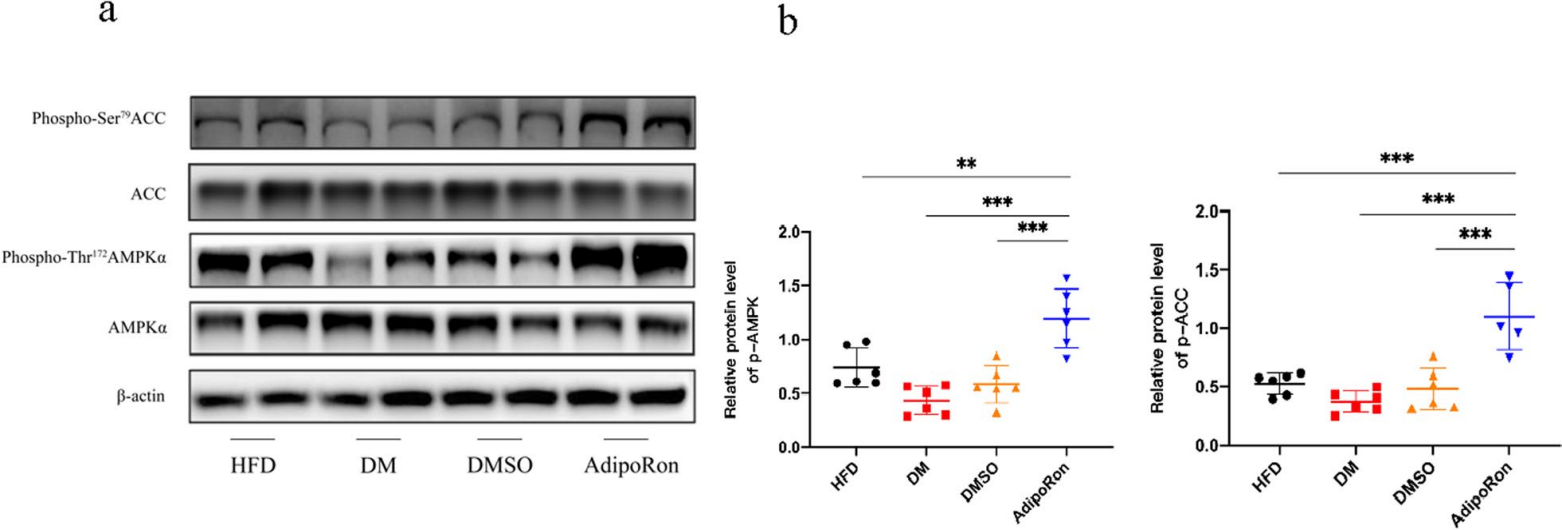
**a**. Altered AdipoR1 pathway-related proteins in the mouse retina. An increase in AMPK and ACC phosphorylation was noticed after AdipoRon treated.ACC and AMPKα remain unchanged, and the expression of p-AMPK and p-ACC is represented by p-AMPK/AMPKα and p-ACC/ACC. **b**: Strips quantified using *Image* *J* software are shown. The results are shown as mean ± standard deviation, **p < 0.01, ***p < 0.001. ACC, acetyl CoA carboxylase; AMPK, 5’ adenosine monophosphate-activated protein kinase; HFD, high-fat diet; DM, high-fat diet + diabetes mellitus control; DMSO, high-fat diet + diabetes mellitus + dimethyl sulfoxide negative control; AdipoRon, high-fat diet + diabetes mellitus + AdipoRon

Phosphorylation of AMPK/ACC is reduced in diabetes. DMSO gavage served as a negative control, and p-AMPK/p-ACC expression was similar to that in the DM group. In contrast, consistent with the AdipoR1 upregulation, phosphorylated AMPK/ACC expression increased synchronously (Fig. 8).

**Fig. 9:**
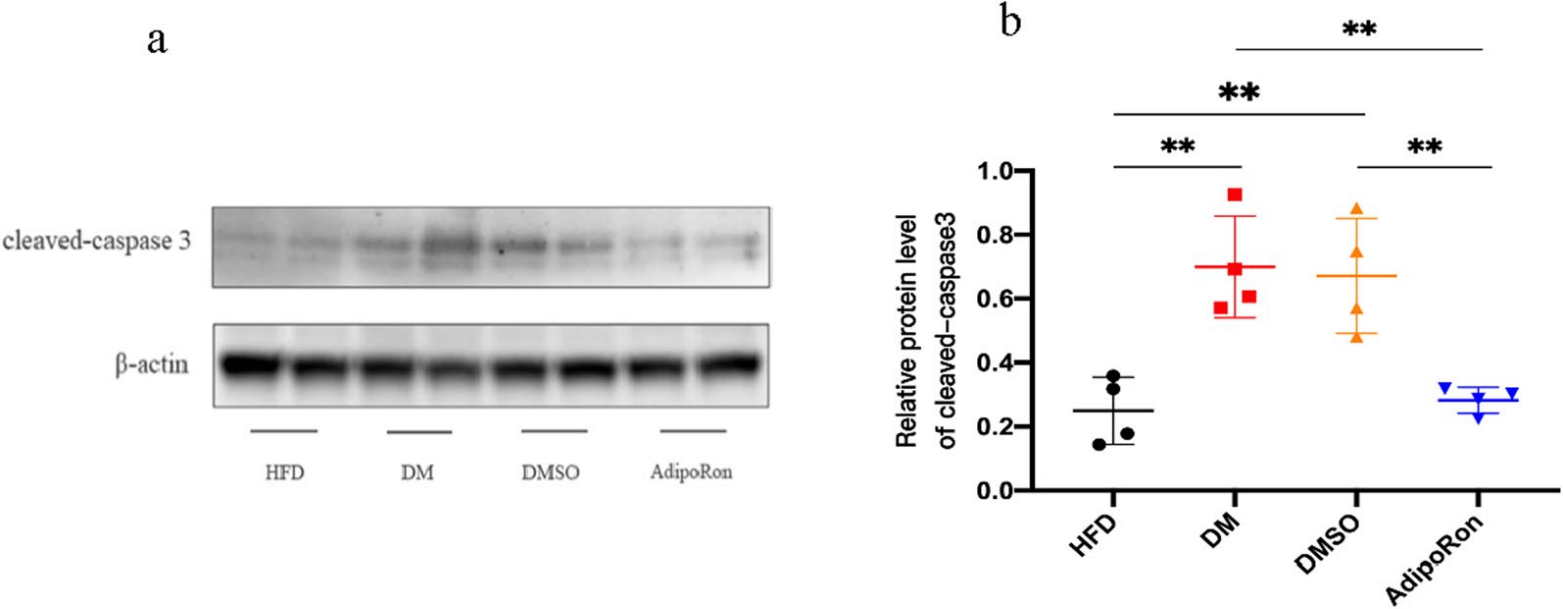
**a**. The expression of cleaved-caspase 3 in the retina of DM and DMSO mice is significantly higher than that of the HFD group and reduced in the AdipoRon group, thereby indicating the alleviation of high-glucose-induced apoptosis after AdipoRon gavage. **b**. Strips quantified using *Image* *J* software. Statistical significance is indicated by ** *p* < 0.01. HFD, high-fat diet; DM, high-fat diet + diabetes mellitus control; DMSO, high-fat diet + diabetes mellitus + dimethyl sulfoxide negative control; AdipoRon, high-fat diet + diabetes mellitus + AdipoRon

Meanwhile, the apoptosis-related protein, cleaved-caspase 3, was increased in the DM and DMSO groups and was reduced in the AdipoRon group, thereby illustrating that AdipoRon exerted an anti-apoptotic effect on the retina (Fig. 9).

### Screening concentrations for adipoRon in vitro

The extracted cells were characterized immunocytochemically with cell-type-specific antibodies (Additional file 1: Figure S1). The survival rates of AdipoRon cells treated with gradient dilutions were detected using CCK-8. At a concentration of 12.5 μM, Müller glia showed the highest survival, resulting in minimal toxicity (Table 2).

**Table 2:** Effect of AdipoRon on Müller glia at different concentrations

AdipoRon concentration (μM)	Survival rates	Inhibition rate
3.125	0.94	0.06
6.25	0.98	0.02
12.5	1.01	− 0.01
25	0.87	0.13
50	0.70	0.30
100	0.50	0.50

### Results of western blot after different treatments

**Fig. 10:**
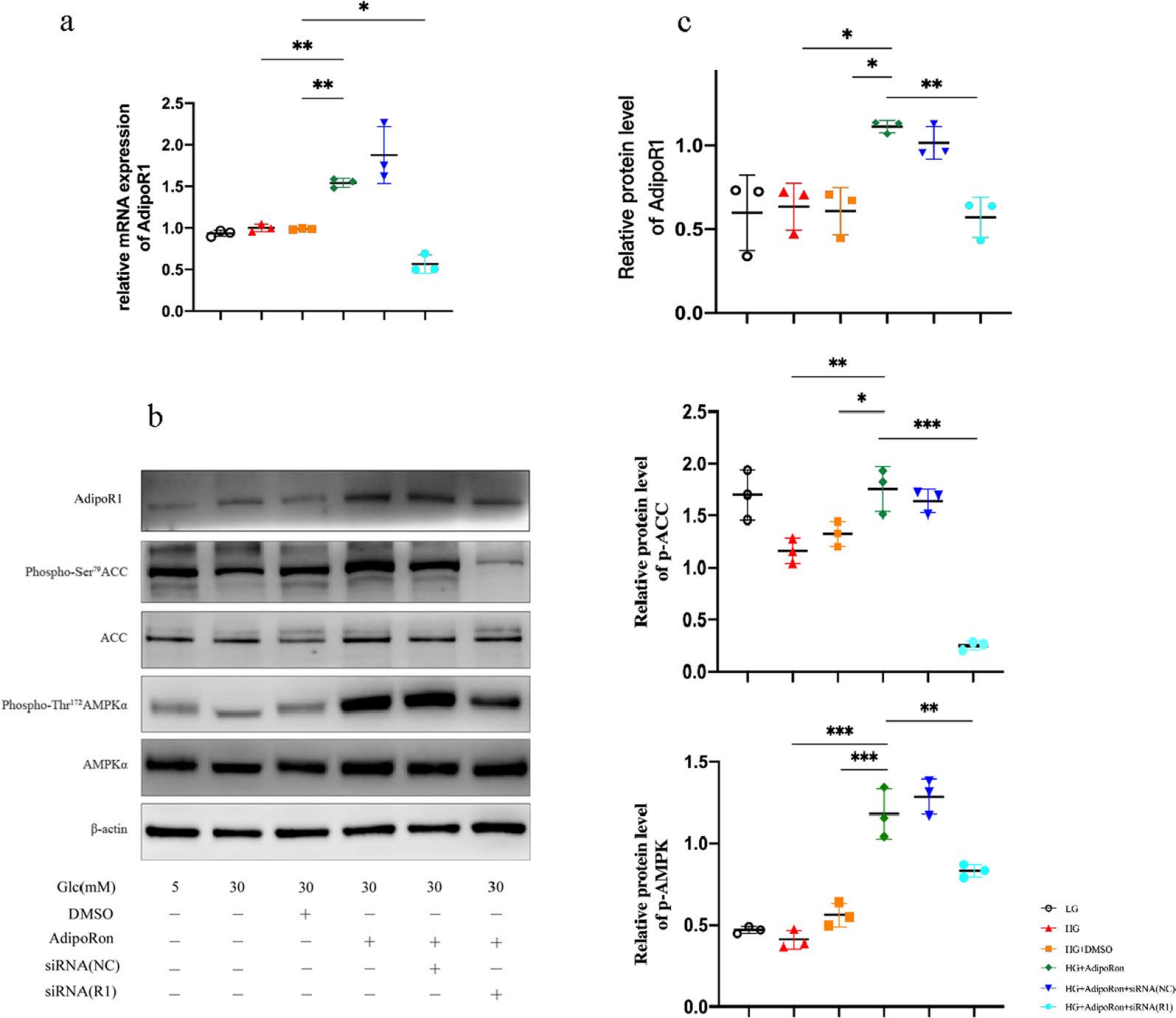
**a**. Quantitative analysis of mRNA expression of AdipoR1 with different treatments. **b**. Changes in the expression of AdipoR1 pathway in Müller glia. Upregulation of AdipoR1 promoted the phosphorylation of the downstream AMPK/ACC and this effect was reversed by the AdipoR1-specific siRNA. ACC and AMPKα are the total proteins, and p-AMPK and p-ACC expression levels are represented by p-AMPK/AMPKα and p-ACC/ACC. **c**. Strips quantified using *Image* *J* software. The results are shown as mean ± standard deviation, *p < 0.05, **p < 0.01, *** *p* < 0.001. **c**. The different expressions of cleaved-caspase 3 among groups. Strips quantified using *Image* *J* software. The results were shown as mean ± standard deviation, **p < 0.01. ACC, acetyl CoA carboxylase; AMPK, 5ʹ adenosine monophosphate-activated protein kinase; AdipoR1, adioponectin receptor 1

The expression of AdipoR1 significantly increased after adding AdipoRon, and there was no significant difference among the low glucose, high glucose, and DMSO groups. After siRNA-NC treatment, AdipoR1 showed no alterations and exhibited high expression. After the siRNA-specific knockdown of AdipoR1, its expression was significantly reduced. The phosphorylation levels of AMPK/ACC in the AdipoRon group were significantly upregulated compared with those in the high glucose and DMSO groups, and the expression of p-AMPK/p-ACC was decreased to a certain extent after siRNA knockdown of AdipoR1 (Fig. 10). The above results showed that the upregulation of AdipoR1 promoted the phosphorylation of the downstream AMPK/ACC pathway, which further verified that AdipoRon mainly acted on Müller glia and induced alterations in downstream pathways through its combination with AdipoR1. For our analysis, the reason why the expression of AdipoR1 was unaltered under high glucose conditions might be related to agonists, glucose concentration, or duration of action. The modification of AdipoR1 was not sufficient to be detected. The changes in AMPK and ACC levels in the high glucose group may be related to the overall state of the cells.

### Oxidative stress levels in different groups

**Fig. 11:**
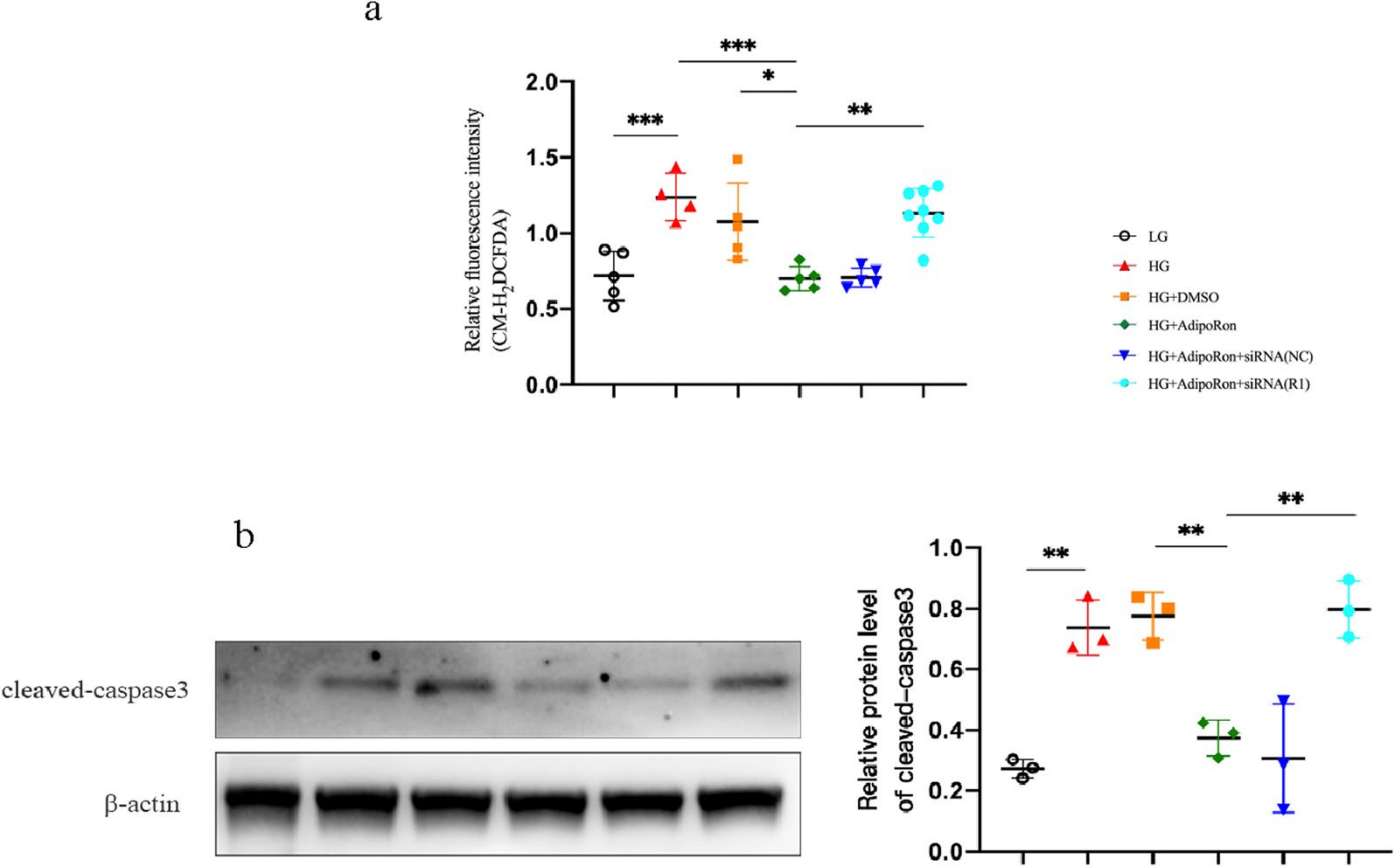
**a**. Oxidative stress levels in each group. The oxidative stress induced by high glucose was alleviated by AdipoRon, and the production of ROS rebounded after the AdipoR1-specific siRNA. **b**. ROS production can be decreased by AdipoRon, following which apoptosis improve. As with oxidative stress, the level of apoptosis increased again after the addition of AdipoR1-specific siRNA. The results are shown as mean ± standard deviation, *p < 0.05, **p < 0.01, ***p < 0.001. *LG* low glucose, *HG* high glucose

Previously, we verified that AdipoRon significantly reduced oxidative stress in the retinas of diabetic mice. Similar changes have been observed in vitro. The oxidative stress level in Müller glia was significantly increased under high glucose conditions and alleviated after AdipoRon was administered. Negative siRNA did not affect oxidative stress, whereas its levels were markedly elevated after targeted depletion of AdipoR1 (Fig. 11). The in vivo studies suggested that AdipoRon played an anti-apoptotic role by activating AdipoR1/AMPK. Similar results were observed in Müller cells in vitro. High glucose-induced apoptosis was reversed by the addition of AdipoRon (Fig. 11).

### Transcriptome sequencing analysis

**Fig. 12:**
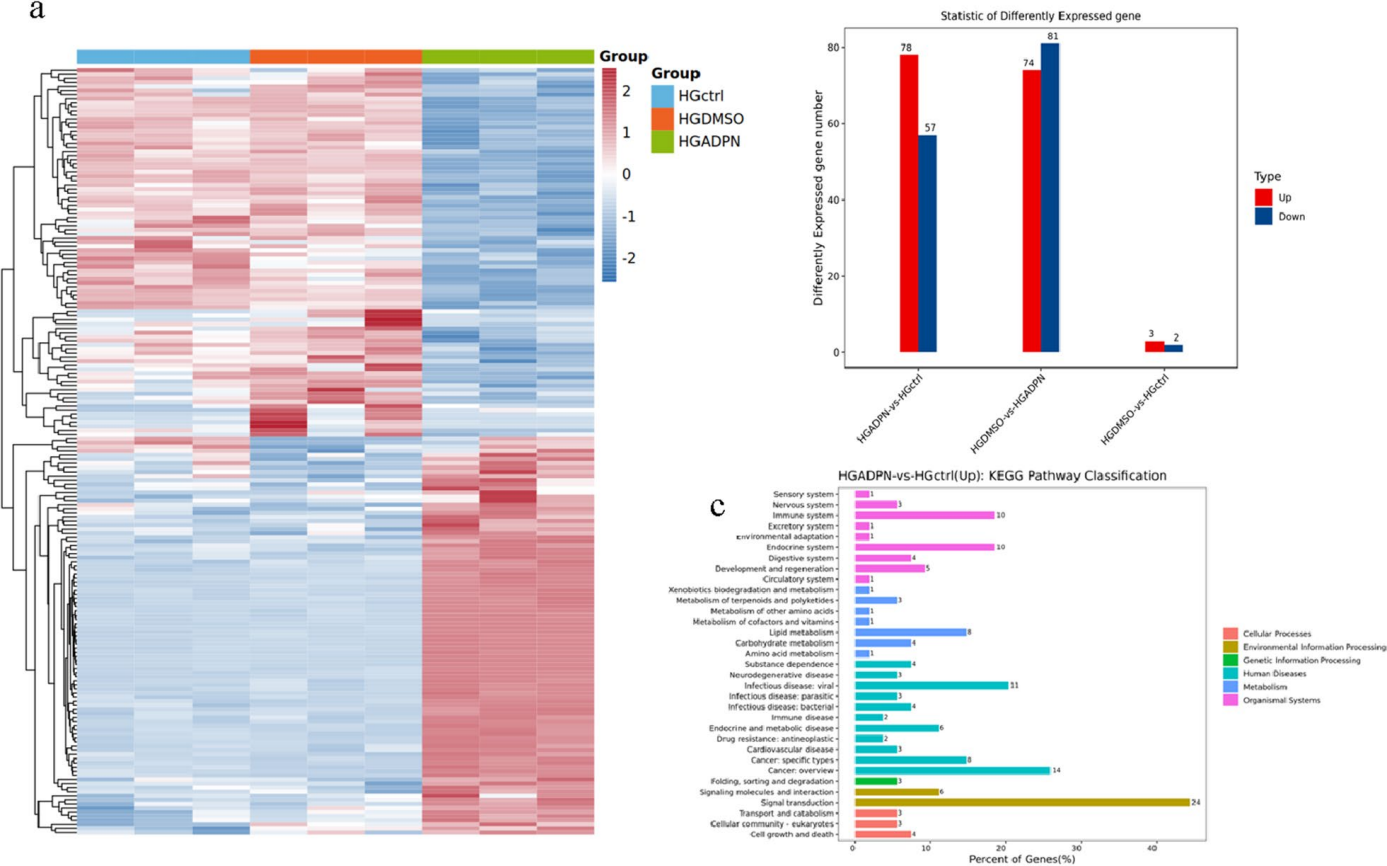
**a**. Heatmap of differential gene expression in three different groups.After the addition of AdipoRon, a number of genes were significantly up-regulated and down-regulated. **b**. Comparison of upregulated and downregulated genes among three groups. **c**. Related pathways involved in genes with significant differences

The Estimate Size Factors of the DESeq (2012) R Package were used to standardize the data, and the p-value and fold-change of the difference comparison were calculated using the nbinom test. Differential transcripts with p < 0.05 and multiple differences greater than 2 were selected for gene ontology and Kyoto Encyclopedia of Genes and Genomes enrichment analysis (Fig. 12).

**Fig. 13:**
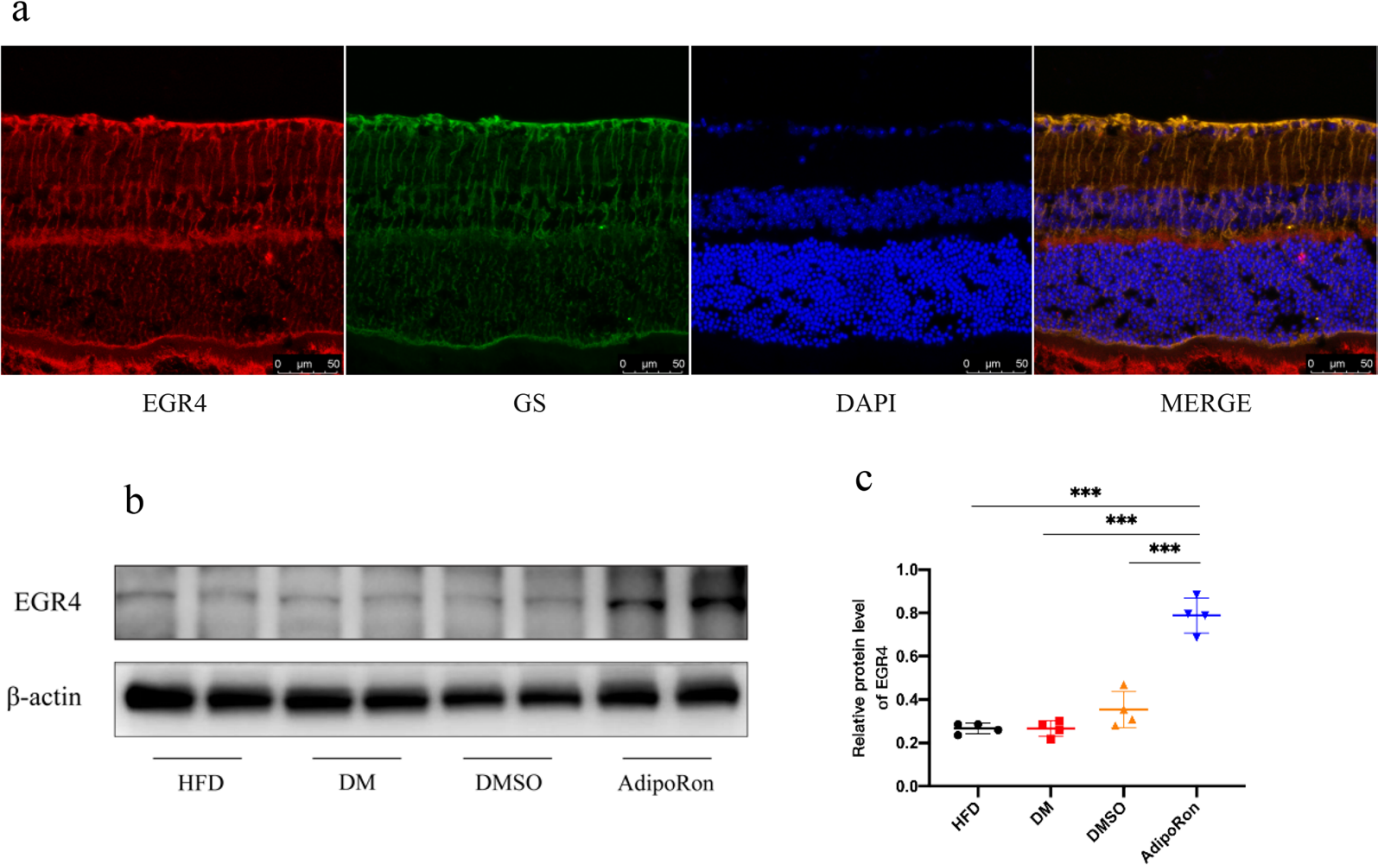
**a**. Retina immunofluorescence staining for EGR4 (red), GS (green), DAPI (blue), and overlay picture.EGR4 was identified to express on Müller cells. **b**. Differences in EGR4 expression in the retinal tissues of mice in different groups. EGR4 showed a tendency of upregulation accompanied with AdipoR1 after AdipoRon gavage. c. Strips quantified with *Image* *J* software. Results are shown as mean ± standard deviation, ***p < 0.001. EGR4, early growth response factor 4; GS, glutamine synthetase; DAPI, 4ʹ,6-diamidino-2-phenylindole; HFD, high-fat diet; DM, high-fat diet + diabetes mellitus control; DMSO, high-fat diet + diabetes mellitus + dimethyl sulfoxide negative control; AdipoRon, high-fat diet + diabetes mellitus + AdipoRon

We conducted multiple comparisons of selected genes, suggesting that EGR4 may be involved in the downstream pathway of AdipoR1 activation. According to transcriptome sequencing results, after AdipoRon intervention, the EGR4 growth factor was approximately 600 times higher than that of other mRNAs, which aroused our interest. However, the corresponding studies are not abundant and this hypothesis would require more exploration.In our study,the expression of EGR4 was confirmed in the retina of normal mice and was located in Müller glia. Western blotting showed that EGR4 expression significantly increased in the retinas of diabetic mice after AdipoRon administration (Fig. 13).

### The in vitro expression and function of EGR4 in the different treated groups

**Fig. 14:**
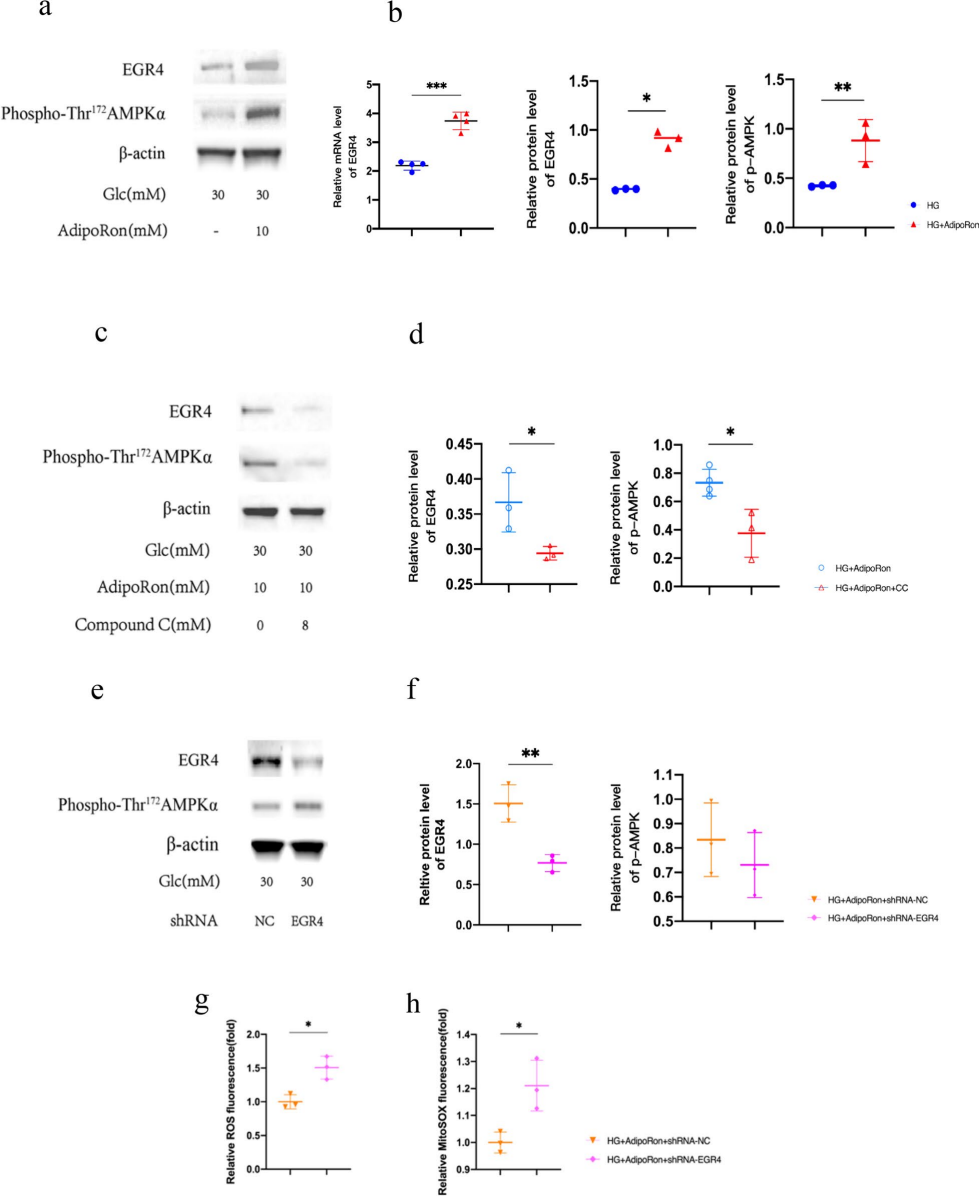
**a**, **c**, **e**. Differences in EGR4 and p-AMPK expression in Müller glia after different treatments.Compound C inhibited p-AMPK and down-regulated EGR4, on the contrary,downregulation of EGR4 did not have an effect on p-AMPK expression. **b**, **d**, **f**. Strips quantified using Image J software. Results are shown as mean ± standard deviation, *p < 0.05, **p < 0.01. **g**, **h**. Total intracellular ROS and mtROS were measured using DCFH-DA probes and MitoSOX probes in Müller by flow cytometry. EGR4 may act both in the nucleus and mitochondria, effectively reduced oxidative stress and protected cellular functions. Results are shown as mean ± standard deviation, *p < 0.05, ** *p* < 0.01. EGR4, early growth response factor 4 AMPK, 5ʹ-adenosine monophosphate-activated protein kinase

To determine whether the in vitro results had the same trend and to thoroughly explore the upstream and downstream regulatory relationships of EGR4 and p-AMPK, we cultured primary Müller cells and silenced EGR4 using shRNA and observed that both EGR4 and p-AMPK were upregulated after AdipoRon treatment. Subsequently, specific inhibitor compound C was added to inhibit AMPK activity, and downregulation of EGR4 and p-AMPK expression was observed. In contrast, we silenced EGR4 using a specific shRNA, and the expression of EGR4 had a significant reduction, whereas that of p-AMPK did not significantly change (Fig. 14). This indicated that AMPK activation was upstream of EGR4 in the pathway. We then confirmed that EGR4 eliminated ROS and mtROS in Müller cells, thereby confirming its role in protecting cells from oxidative stress. Considering the ROS and mtROS results, it was reasonable to assume that EGR4 decreased cellular ROS levels by reducing ROS biogenesis in mitochondria. This suggested that the EGR4 transcription factor, which may act both in the nucleus and mitochondria, effectively reduces oxidative stress and protects cellular functions. Although we had trouble confirming how it acts on the mitochondrial respiratory chain, these findings may provide clues to new mechanisms (Fig. 14).

## Discussion

The oral agonist AdipoRon used in this study is an endogenous adiponectin analog and a small-molecule agonist that was successfully identified by Okada in 2013. Additionally, it can highly mimic the biological effects of adiponectin, binding to specific receptors and activating intracellular signaling pathways to exert physiological activity. In this study, AdipoRon was orally administered to STZ-induced diabetic mice, and relevant experimental data revealed that AdipoRon alleviated oxidative stress and apoptosis of the retina by activating the AdipoR1/AMPK/ACC pathway, improved retinal function, and reversed retinal damage caused by long-term hyperglycemia and hyperlipemia. Furthermore, differentially expressed mRNAs were identified by transcriptome sequencing in the AdipoRon-treated group. Among these, EGR4 may have a potential protective effect on DR.

Diabetes causes a rise in plasma glucose, TG, and NEFA, indicating a glycolipid metabolism disorder. Since decreasing serum lipids could reduce the risk of developing DR [42], the final gavage dose was determined by pre-experiments, which did not affect plasma lipid levels during intragastric administration; however, it upregulated the expression of AdipoR1. The study demonstrated that AdipoRon might not protect the retina by lowering plasma glucose or lipid levels, consistent with the previous studies [18, 24, 43]. The first possibility is that the glucose-lowering action of AdipoRon was transient instead of long-term stabilization. The second was that the dose of AdipoRon was insufficient to affect glucose homeostasis; however, it was sufficient to improve cellular function. Therefore, it was preliminarily determined that AdipoRon improved retinal function by reducing plasma glucose or lipids alone and by exerting biological effects through binding to specific receptors, which further activated signaling pathways.

In this study, we observed an increase in ERG a and b wave amplitude, a significant decrease in Müller glia reactive hyperplasia, and a recovery of RGC quantity in the AdipoRon-gavaged group. Although the agonist played a role in combination with Müller, it was gradually confirmed that the elongated Müller closely interacted with other types of cells in the retina [44] and the neurovascular unit [45] structure was the core of DR pathogenesis. Extracellular glutamate is transported to Müller cells by glutamate transporters and converted into non-toxic glutamine. The released glutamine is then taken up by neurons and hydrolyzed by glutaminase to form glutamate. If the transport of Müller cells is impaired and circulation stops, neuronal function will deteriorate rapidly [46]. Müller cell processes enclose cell bodies and processes in retinal neurons. Considering that Müller cells and neurons are structurally tightly connected, it helps maintain neuron function by providing energy substrates and neurotransmitter precursors to nerve terminals [47]. In recent years, there has been growing evidence that disruption of Müller cell metabolism results in neuron death and RGC apoptosis [48–51]. These complex interactions between retinal cells lead to alterations in retinal function.

Many possible mechanisms could explain the apoptosis of retinal cells; however, oxidative stress is one of the most important, and AdipoRon was proven to have a significant effect. Excessive accumulation of ROS, mainly from the mitochondrial respiratory chain, leads to oxidative stress and even lipid peroxidation, damaging retinal cells, nerves, and vessels [52]. In the retina, the neurons and the outer segment of the retina have the highest density of mitochondria, which explains why the location where the fluorescence intensity changed in our DHE was mainly in the inner layer of the retina and retinal pigment epithelium. Similar conclusions have been reported previously by Tonade [53]; diabetes increases oxidative stress in the outer layer of the retina and could be reversed by dietary supplementation with antioxidants, suggesting that the efficacy of AdiopRon is related to the improvement of oxidative stress, especially mitochondrial dysfunction.

Western blotting further confirmed that the expression of AdipoR1 in the retina of AdipoRon-treated diabetic mice was significantly increased, similar to the phosphorylation of AMPK/ACC. Many studies have shown that adiponectin levels are decreased in patients with diabetes; however, an opposite increasing trend is exhibited in the retina. This is because of the presence of the blood-ocular barrier; hence, adiponectin in the circulation was observed to be inconsistent with that in the eyes. Although it was premature to conclude whether the local adiponectin increase was gathered from the circulation or via autocrine pathways, it was hypothesized that this increase may be related to compensatory upregulation in response to the adverse environment of the retina, which finally caused an increase in AdipoR1 expression. This complied with our results that AdipoR1 expression was slightly increased in DM and DMSO group mice. However, intracellular phosphorylation of AMPK/ACC was not detected, suggesting that the compensatory upregulation of AdipoR1 could not effectively activate the corresponding intracellular pathway. Alternatively, the compensatory upregulation of adiponectin and AdipoR1 tended to decrease with the progression of DM; however, the specific mechanisms need further exploration and verification. Sustained oxidative stress caused apoptosis, and cleaved-caspase 3 expression was confirmed to be significantly reduced after AdipoRon administration in diabetic mice, suggesting a certain degree of reversal of retinal apoptosis.

AMPK is a key protein in signal transduction pathways, phosphorylates various downstream transcription factors, and regulates glucose metabolism, lipid metabolism, cell proliferation, and other processes. The AdipoRon-activated AMPK/unc-51 like autophagy activating kinase 1 pathway in HK-2 cells inhibited epithelial mesenchymal transformation by promoting AMPK-mediated autophagy activation [54]. Uchida [55] discovered that ROS from retinal ganglion cells, which were incubated with 10 µM of AdipoRon, inhibited by improving mitochondrial metabolism and reducing oxidative stress, which ultimately had a protective effect on the optic nerve. In this study, we identified Müller glia as the cell that specifically interacted with AdipoRon, and the optimal AdipoRon concentration determined using CCK-8 was 12.5 μM. In an AdipoRon-treated high glucose environment, intracellular AdipoR1/AMPK/ACC was upregulated. Subsequently, AdipoR1 knockdown, mediated by siRNA, reduced ROS and apoptosis levels induced by high glucose, consistent with our in vivo experiments. This might be related to the inhibition of AMPK activation. Emerging research has shown that AMPK is a redox sensor and modulator, playing pivotal roles in disease progression. The AMPK-PGC1α pathway in human umbilical vein endothelial cells was identified to promote superoxide dismutase induction and mitochondrial biosynthesis [56]. Moreover, AMPK regulates mitochondrial fission through the autophagy-dependent degradation mechanism of dynamin-related protein 1 [57]. In addition, miglitol inhibited apoptosis and mitochondrial superoxide production by activating the AMPK-endothelial nitric oxide synthase axis, thereby protecting endothelial cells from oxidative stress damage [58]. Inhibiting the expression of ROS-producing enzymes, such as nicotinamide adenine dinucleotide phosphate (NADPH) oxidase activation by AMPK, was another efficient way to alleviate oxidative stress [59]. As a key signaling molecule, AMPK might have a large protein family downstream that plays a role in various physiological activities.

Combined with transcriptome sequencing results and a literature review, we concluded that EGR4 might be a key protein after AdipoR1 activation. EGR4 is a transcription factor belonging to the EGR family and is involved in signal transduction, tumorigenesis, and immune response [60]. EGR4 plays an important role in neuronal maturation in the central nervous system [61]. Mengozzi [62] discovered that EGR2 and EGR4 were upregulated by neuroprotective erythropoietin in a model of middle cerebral artery occlusion, and this regulation was validated in stroke-related experiments, highlighting their association with ischemia. Niu [63] reported that pathological changes caused by cerebral ischemia were reversed after the injection of the EGR4 adenovirus into the brain. In this study, we verified that EGR4 was expressed in Müller glia and increased significantly after AdipoRon administration. The reduction of ROS and MitoSox suggested that its effects were closely related to oxidative stress, specifically mitochondrial oxidative stress. The mechanism of action of EGR4 is less well studied. As a member of the EGR family with a zinc finger structure, many related mechanisms were possible. EGR1 activation in H9C2 cells was mitogen-activated protein kinase/extracellular signal-regulated kinase, and c-Jun N-terminal kinase-dependent [64]. Moreover, ChIP assays revealed that EGR1 was a transcriptional activator of NADPH oxidase 4, a key oxidative stress enzyme in diabetic kidney disease [65]. We hypothesized that the protective effects of EGR4 mentioned previously were achieved by reducing oxidative stress or mitochondrial oxidative stress. The mechanism of action may be related to promoting the transcription of many nuclear coding components in the electron transport chain. Important regulators of mitochondrial biosynthesis were closely related to PGC-1α [66]; downstream of AMPK, regulation of EGR4 by PGC-1α remained unclear. Alternatively, these two proteins belong to two independent pathways, thereby collectively contributing to cellular oxidative stress reduction. Hence, this was the next step in our investigation

From the above discussion, it was concluded that dual activation of AMPK and EGR4 was important for AdipoRon to achieve cellular and retinal protection in a high-glucose environment. However, the exact mechanism of action merited further research.

These results revealed an entirely new participant in the intracellular pathway activated by AdipoR1. In this study, the retinal protective effect of AdipoRon was verified in vitro and in vivo for the first time. We demonstrated that AdipoRon activated classical AMPK/ACC pathways by binding to AdipoR1 on Müller glia. Moreover, transcriptome sequencing provided a clue for further mechanism research. Furthermore, inhibition of AMPK downregulated the expression of EGR4 and revealed it as a key downstream factor of AMPK via transcriptional control. However, reports on the action of EGR4 in DR are limited. We hypothesized that EGR4 reduced oxidative stress by maintaining mitochondrial homeostasis, ultimately reducing cell apoptosis. Further exploration of additional specific pathways is warranted and may provide new potential therapeutic targets.

## Conclusions

AdipoRon, a small-molecule agonist, activated the AMPK/ACC pathway by specifically binding to AdipoR1 on Müller glia, thereby improving oxidative stress and apoptosis of retinal cells and tissues and retinal protection from high glucose. AdipoRon was found to upregulate EGR4 by activating pathways independent of AMPK, and it is expected to provide new ideas for treating DR.

### Supplementary Information

**Additional file 1: Figure S1.** Cell were characterized immunocytochemically with cell-type-specific antibodies,that is ,KIR4.1,GS. KIR4.1,Inwardly rectifying potassium channel subtype 4.1; GS, Glutamine synthetase; RBPMS, RNA-binding protein with multiple splicing; IBA-1,ionized calcium binding adapter molecule 1.

## Data Availability

The authors declare that all other data supporting the findings of this study are available within the article and its Supplementary Information files, or are available from the corresponding author upon reasonable request.

## Abbreviations

AdipoRs: Adioponectin receptors
DM: Diabetes mellitus
DR: Diabetic retinopathy
PDR: Proliferative diabetic retinopathy
RGCs: Retinal ganglion cells
AMPK: 5ʹ Adenosine monophosphate-activated protein kinase
ACC: Acetyl CoA carboxylase
TG: Triglyceride
NEFA: Non-esterified fatty acid
GS: Glutamine synthetase
GFAP: Glial fibrillary acidic protein
ROS: Reactive oxygen species
STZ: Streptozotocin
EGR4: Early growth response protein 4
PPAR: Peroxisome proliferators-activated receptor
NADPH: Nicotinamide adenine dinucleotide phosphate
RBPMS: RNA-binding protein with multiple splicing
PGC: Peroxisome proliferator-activated receptor-gamma coactivator
siRNA: Small interfering RNA
DAPI: 4ʹ,6-Diamidino-2-phenylindole
DMSO: Dimethyl sulfoxide
ERG: Electroretinogram
PBS: Phosphate-buffered saline
BSA: Bovine serum albumin
P/S: Penicillin–streptomycin
TBST: Tris-buffered saline with Tween 20
DMEM: Dulbecco's modified eagle medium
CCK-8: Cell Counting Kit-8
CM-H2DCFDA: 5,6-Chloromethyl-2ʹ,7ʹ-dichlorodihydrofluorescein diacetate
shRNA: Short hairpin RNA
mtROS: Mitochondrial ROS
HBSS: Hanks’ balanced salt solution
SD: Standard deviation
HFD: High-fat diet
DHE: Retinal oxidative stress detection
