# Supplementary material for: Small-molecule agonist AdipoRon alleviates diabetic retinopathy through the AdipoR1/AMPK/EGR4 pathway
Source: J Transl Med. 2024 Jan 2;22:2. doi: 10.1186/s12967-023-04783-3 (PMC10759471; doi:10.1186/s12967-023-04783-3)
Supplement: Supplementary file 1 — Additional file 1: Figure S1. Cell were characterized immunocytochemically with cell-type-specific antibodies,that is ,KIR4.1,GS. KIR4.1,Inwardly rectifying potassium channel subtype 4.1; GS, Glutamine synthetase; RBPMS, RNA-binding protein with multiple splicing; IBA-1,ionized calcium binding adapter molecule 1. [file 12967_2023_4783_MOESM1_ESM.docx]

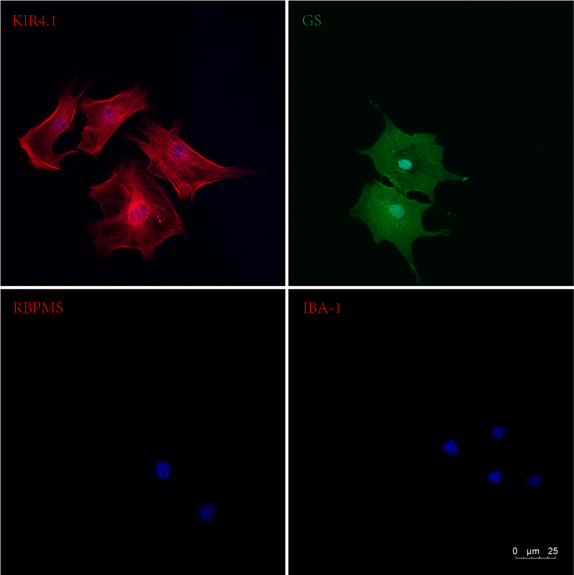


Figure S1 Cell were characterized immunocytochemically with cell-type-specific antibodies,that is ,KIR4.1,GS. KIR4.1,Inwardly rectifying potassium channel subtype 4.1; GS, Glutamine synthetase; RBPMS, RNA-binding protein with multiple splicing; IBA-1,ionized calcium binding adapter molecule 1.
